# Network evolution model for supply chain with manufactures as the core

**DOI:** 10.1371/journal.pone.0191180

**Published:** 2018-01-25

**Authors:** Haiyang Fang, Dali Jiang, Tinghong Yang, Ling Fang, Jian Yang, Wu Li, Jing Zhao

**Affiliations:** 1 Department of Military Logistics, Army Logistics University, Chongqing, China; 2 Department of Mathematics, Army Logistics University, Chongqing, China; 3 Department of Military POL, Army Logistics University, Chongqing, China; 4 Institute of Interdisciplinary Complex Research, Shanghai University of Traditional Chinese Medicine, Shanghai, China; Georgia Institute of Technology, UNITED STATES

## Abstract

Building evolution model of supply chain networks could be helpful to understand its development law. However, specific characteristics and attributes of real supply chains are often neglected in existing evolution models. This work proposes a new evolution model of supply chain with manufactures as the core, based on external market demand and internal competition-cooperation. The evolution model assumes the external market environment is relatively stable, considers several factors, including specific topology of supply chain, external market demand, ecological growth and flow conservation. The simulation results suggest that the networks evolved by our model have similar structures as real supply chains. Meanwhile, the influences of external market demand and internal competition-cooperation to network evolution are analyzed. Additionally, 38 benchmark data sets are applied to validate the rationality of our evolution model, in which, nine manufacturing supply chains match the features of the networks constructed by our model.

## Introduction

**Fig 1:**
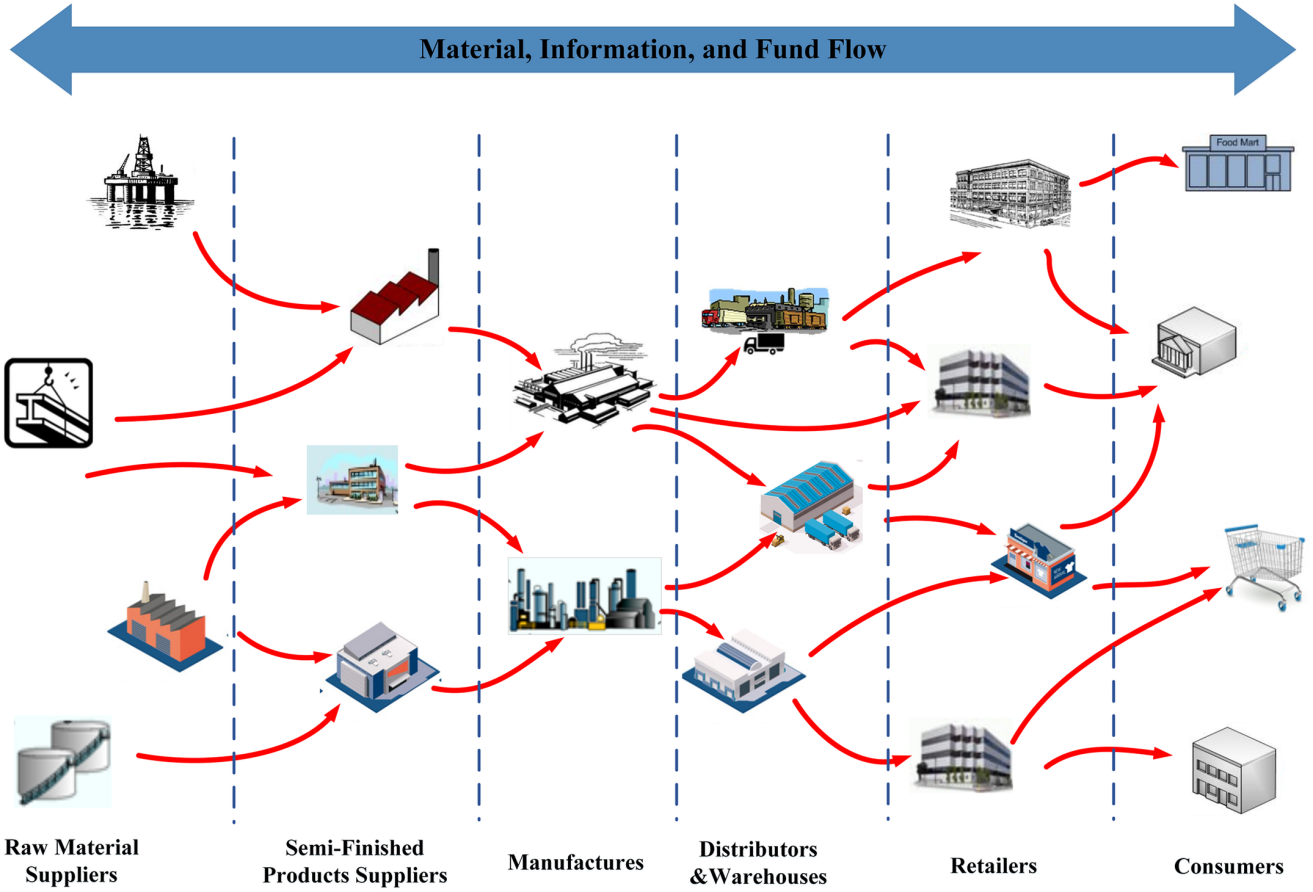
The schematic diagram of supply chain network, which is a network with manufactures as the core.

The supply chain network consists of overwhelming number of enterprises and their mutual partnerships, in which the enterprises cooperate with the core enterprises directly or indirectly [1–3]. In such a network, enterprises are nodes and partnerships (In this work, the partnership means supply-demand relationship) among them are links. As shown in Fig 1, the supply chain network with manufactures as the core controls material flow, information flow and value flow from the process of raw material purchasing, through the processes of semi-finished products processing, manufacturing, warehousing, distribution, and finally to retail process that sells products to consumers[4–7]. It has most common characteristics as other complex networks, such as large scale, sparse connection, small world, scale-free, dynamic, self-similarity, and super family [8–12]. The most specific feature of supply chain network is multi-hierarchical, in which enterprises are both demand-sides and supply-sides, while those in the same layer have similar businesses and functions[13, 14]. Meanwhile, the supply chain network always changes in order to adapt external market demand and internal competition-cooperation. Hence, enterprises with strong competitiveness will become stronger and their partner numbers will also increase. On the contrary, weaker enterprises may be eliminated and replaced by other new members for impeding supply chain development[15, 16].

Understanding the evolution law of supply chain network can help us to manage and control it. Surya D. Pathak pointed out that the supply chain is a typical complex adaptive system. He studied the dynamic evolution and growth of supply and demand network and discussed whether there exist some simple rules that control its emergence and growth [17]. H.-J. and J.-J. Wu quantitatively analyzed the scale-free property of real supply chain networks in the process of evolution [11]. Dirk Helbing found that the complexity of supply chain cannot be explained by traditional algorithms or models, and came to the conclusion that the topology and macro nature of the supply chain network directly affect its micro changes [18]. Besides that the internal competition-cooperation plays important role in the process of evolution, the competitions among supply chains also exist. For example, the two supply chains that provide some type of products may compete against each other for acquiring the limited market share. Another example is that an enterprise supplies raw materials to multiple supply chains, which may also cause the competition among them. Hence, the competitions among supply chains are also important for their development [19–21]. We understand that the study of the effect mechanism about internal competition-cooperation to the evolution of the supply chain is the foundation and premise for studying about the competition among multiple supply chains systems. Thus the focus of our work is on the research about the effect of competition-cooperation to the evolution of a single supply chain.

Currently, there are two methods for analyzing the evolution of supply chain. The first method study the evolution law of competition-cooperation among the enterprises in the supply chain based on the game theory [22–25]. The current studies of such method is only used to the analysis of supply chain with simple structure and less enterprises. For the complex supply chain, this method is no longer applicable. The second method to understand the evolution law is to build evolution models according to the network topology structure and inherent property of the supply chain. The earliest evolution model is based on the BA evolution model of scale-free networks, which follows the rules of preferential choice and dynamic growth [26]. However, structures of networks created by such evolution model are far from that of real supply chain networks. Therefore, later studies integrated some specific information of supply chain to the original evolution model. In view of the elimination of enterprises, Ref. [27] randomly deleted nodes and used the principle of preferential choice to compensate missing edge caused by the loss of nodes. Since the increase rate of the supply chain scale is inhomogeneous, and the network topological structure will be broken by the addition of new nodes, Ding *et al* built an evolution model that considers the effect caused by individual choice behavior of enterprise nodes [28]. However, this model ignores the effect of transaction cost to the link between two nodes, thus they applied the theory of weighted networks to study the evolution of supply chain network, in which, the weights of edge represent the transaction costs among the enterprise nodes [29]. Liu *et al* considered the whole macroscopic behavior of supply chains networks, analyzed growth evolving rule of birth, decline, and exit of enterprise nodes, and constructed a layered weighted supply chain network model based on preferential attachment measured by the combination of multi-attribute parameters [30]. Sun *et al* put forward a five- level local world network model. This model used the BA model and the multi-local world theory as the foundation, combined with the reality of network node generation and exit mechanism. The topology structure of networks constructed by this model is similar to that of the real supply chain [31]. To measure the risk effect to the supply chain network, *Ref* [32] defined the transaction risk as the distance between the two enterprise nodes in the evolution model. Fu *et al* thought that the rule of preferential choice is decided not only by the node degree or strength, but also the path length between two enterprise nodes in the real clustered supply chain network [33]. Similar viewpoint was also held by Cao *et al*. In their work, the weight is defined as cooperation benefit between two enterprises [34]. Gang *et al*. introduced a topological model for supply networks, in which repeated prisoner’s dilemma game is applied. The result shows that heterogeneous structures are helpful to promote cooperation, and strategy payoff and competition pressure are also involved in the evolution of cooperation [35].

Although the evolution of supply chain network has been widely studied, the existing models have some shortages. First, most current evolution models of supply chain are based on evolution model of complex network[26]. However, the structures between common network and real supply chain exist great difference. The supply chain network is a type of hierarchical network. Since the edge denotes the relationship of supply-demand, the nodes in the same layer do not build partnership. Despite some works considered the hierarchical structure, the cooperation of enterprises is only between neighbor hierarchies, but not cross layers[31]. Second, only a few studies have considered the impact of external market demand on supply chain development in evolution model[36, 37]. In fact, the supply chain development is closely associated with the external market demand. Once the external market demand changes, the supply chain will adjust itself in order to adapt it. Third, the development process of three stages and flow balance of supply chain are not considered in previous evolution models. Similar as ecosystem, supply chain development also follows the rule of survival of the fittest, that is to say, the network scale obeys Logistic function. Moreover, the supply and demand in the whole supply chain network system are conservative. When the enterprise (or partnership) addition (or deletion) occurs, the conservative will be broken. For seeking benefits, the whole system and individuals will restore new balance quickly. Hence the network flow balance should also be considered.

In this paper, to overcome the shortages of previous evolution models, we propose a new evolution model, which simulates the evolution of supply chain with manufacturers as the core based on evolutionary theory and BA evolution model. The supply chain with manufacturers as the core is acyclic and does not involve self-loop, feedback, and closed-loop. Our model considers the pulling of external market demand and the pushing of internal competition-cooperation. Compared with the traditional evolution models, it considers the flow conservation, which further makes the model in consistence with the supply chain development. Meanwhile, in order to illustrate the rationality of the model, the bench-mark data of supply chain are applied to validate the networks created by our evolution model.

## Methods

### Supply chain network related concepts

In our study, the supply chain network is defined as a directed weighted network *G* = (*V*, *E*, *W*), where *V* is the set of all the enterprise nodes, *E* is the set of all edges, and *W* is the set of all weights. The edge *e*_*ij*_ ∈ *E* represents the partnership from node *v*_*i*_ to *v*_*j*_, and *w*_*ij*_ ∈ *W* is the weight of *e*_*ij*_, which represents the cooperation strength between two enterprises. In order to facilitate subsequent description, we assume *w*_*ij*_ = 0, if *e*_*ij*_ ∉ *E*.

Before giving the relevant definitions, we need to make an assumption on the network hierarchy. Generally, enterprises in the same layer have the same local world, similar network connections, and similar core businesses. One enterprise in the supply chain only belongs to one layer. Specifically, we suppose that the aforementioned supply chain network *G* = (*V*, *E*, *W*) has *L* layers, which are divided according to the functions and attributes of the nodes. If we define all the nodes in the same layer *I* as the set ${\tilde{H}}_{I}$, *I* = 1, 2, ⋯ *L*, the following formula holds: $\overset{L}{\underset{I=1}{\cup }}{\tilde{H}}_{I}=V$, and ${\tilde{H}}_{I}\cap {\tilde{H}}_{J}=\emptyset$.

In the following, some definitions related to supply chain network are introduced for clearly expressing our evolution model.

#### Definition 1: Layer-layer association probability

In a multi-layer supply chain network, we define layer-layer association probability as the likelihood of existing cooperation between enterprise nodes in the layer *I* and layer *J*, and denote it by $p_{IJ}^{\left(h\right)}$, where *h* presents layer. Layer-layer association probability is determined by priori information of supply chain’s property, which is probabilistic and fixed. This definition overcomes the shortages of previous studies, which cannot measure the cooperation of two enterprises, which are not located in adjacent layers. [38]. Obviously, $p_{IJ}^{\left(h\right)}=p_{JI}^{\left(h\right)}$.

#### Definition 2: Layer-layer cooperation strength

Given a multi-layer supply chain network, layer-layer cooperation strength is defined as the sum of the weights for all the partnerships between the two layers, and associated with the flow of the supply chain. More specifically, let $q_{IJ}^{\left(h\right)}(t)$ denote layer-layer cooperation strength between layer *I* and *J* at the time *t*, then we have

$$q_{IJ}^{\left(h\right)}(t)=\sum_{v_{i}\in {\tilde{H}}_{I}\&v_{j}\in {\tilde{H}}_{J}\&(v_{i},v_{j})\in E}\left(w_{ij}(t)+w_{ji}(t)\right)$$ (1)

where *w*_*ij*_(*t*) is the weight of edge *e*_*ij*_ at the time *t*, and $q_{IJ}^{\left(h\right)}(t)=q_{JI}^{\left(h\right)}(t)$.

#### Definition 3: Layer node weight

In the multi-layer supply chain, layer node weight indicates the importance of corresponding enterprise in its layer. It is determined by the enterprise’s competence and its cooperation strength. Layer node weight $c_{i}^{\left(h\right)}(t)$ of node *v*_*i*_ could be calculated as follows:

$$c_{i}^{\left(h\right)}(t)=\alpha_{c}\frac{c_{i}^{\left(n\right)}(t)}{\sum_{v_{j}\in H_{i}}c_{j}^{\left(n\right)}(t)}+(1-\alpha_{c})\frac{\sum_{v_{j}\in V}w_{ij}(t)}{\sum_{v_{k}\in H_{i}\&v_{j}\in V}w_{kj}(t)}$$ (2)

where $c_{i}^{\left(n\right)}(t)$ is the enterprise competence of node *v*_*i*_ at the time *t*, which represents the competitiveness of enterprise *v*_*i*_ in its industry; constant *α*_*c*_ is the weight of enterprise competence and 0 ≤ *α*_*c*_ ≤ 1. The initial enterprise competence is generated randomly. *H*_*i*_ denotes the set of all nodes in the layer that node *i* located.

It should be noted that, layer node weight of new enterprise node is only determined by its competence, because the enterprise has not established any cooperation in the network. In other words, we should take the weight *α*_*c*_ = 1 in this case.

#### Definition 4: Matching coefficient

Matching coefficient indicates the difference between two enterprises. Assuming that node *v*_*i*_ is in layer *I* and node *v*_*j*_ is in layer *J*, matching coefficient is defined as follows:

$$z_{ij}(t)=\text{exp}\left(-\left|\frac{\left\|{\tilde{H}}_{I}\right\|c_{i}^{\left(h\right)}(t)}{\sum_{v_{k}\in {\tilde{H}}_{I}}c_{k}^{\left(h\right)}(t)}-\frac{\left\|{\tilde{H}}_{J}\right\|c_{j}^{\left(h\right)}(t)}{\sum_{v_{k}\in {\tilde{H}}_{J}}c_{k}^{\left(h\right)}(t)}\right|\right)$$ (3)

where ||·|| represents the number of elements in the set.

Actually, the enterprise would rather cooperate with outstanding entities. However, the paradox lies in that outstanding enterprises tend to cooperate with each other. Matching coefficient shows the difference between the important extents of the two enterprises in their own layers. The less the difference, the larger the probability that they build cooperation.

### Evolution rules

The system of the whole supply chain is similar with the ecosystem in nature, which follows the rule of survival of the fittest [39]. Under the open market, the existing purpose of supply chain is to seek benefits for the enterprises through adapting to the external market demand [40]. Thus, the fluctuation of external market demand will cause the evolution of supply chain. Meanwhile, within the bounds of the contracts, the enterprises in different layers are relatively independent, and they will attempt to pursuit the maximized efficiency or benefit in a particular period. This behavior is the force that drives supply chain to adapt external market demand, and also the internal factor that promotes the evolution of supply chain. Based on the above analysis, this paper will propose evolution rules that could describe the evolution of supply chain from the perspective of the inducement of external market demand and the promotion of internal competition-cooperation.

#### (1) External market demand

External market environment includes a number of factors, which include the changes of market conditions or policy, country-level sanctions, natural disasters, outbreaks of wars or riots, and others. These factors are much important to the evolution of the supply chain and they can divide into two types, which are steady state disturbance and abrupt disturbance. The factors of abrupt disturbances may cause the mutation of supply chain, and the evolution law of supply chain will change, so this work do not consider abrupt disturbance. Therefore, only relatively stable factors of the external market environment are studied, and then those factors are simplified as the change of external market demand. Here, we propose two evolution rules to describe how the supply chain adapts external market demand.

**Rule 1: The scale and flow of supply chain satisfy stable power law relation**. The supply chain scale, also called asset scale, is the asset stock of the whole supply chain network. It can ensure the management of the supply chain normally. Theoretically, the supply chain scale should be reasonable. The scale is too large, the resources will be idle and the turnover of funds will be slow. Conversely, it will be difficult to meet the needs of market operation. The supply chain flow includes information flow, material flow, fund flow, and others. Generally, we use fund flow to measure the supply chain flow, since it can be measured easily. The size of the supply chain flow is decided by the market demand directly. Meanwhile, the flow is the main reason that affects the supply chain stability. *Ref*. [41] analyzed data of global top 2000 companies from 2007–2009 Forbes magazine, and concluded that the scale and the flow of the enterprises (materials, retail, durable goods manufacturing, etc.) satisfy stable power law relation. In fact the supply chain scale *M*(*t*) also obeys power law [42, 43], that is:

$$M(t)={\left(F(t)/F_{0}\right)}^{\frac{1}{\lambda }}$$ (4)

where, *M*(*t*) represents the supply chain scale at the time *t*. Since the power law distribution of supply chains for different types of industries are various, and the supply chain studied in this paper is with manufactures as the core, thus the *λ* is set as 0.818 according to *Ref*. [41]. *F*(*t*) is the supply chain flow. *F*_0_ denotes constant of flow. According to *Ref*. [44], the change of supply chain flow with time *t* is described by *Logistic-increasing* model as follows:

$$\frac{dF(t)}{dt}=\left(a-bF(t)\right)F(t)+\varepsilon_{F}(t)$$ (5)

where, *a* denotes natural growth rate of the supply chain flow; *b* is the retardation coefficient of the flow, and *ε*_*F*_(*t*) is white noise at the time *t*, and the amplitudes of it is fixed.

**Fig 2:**
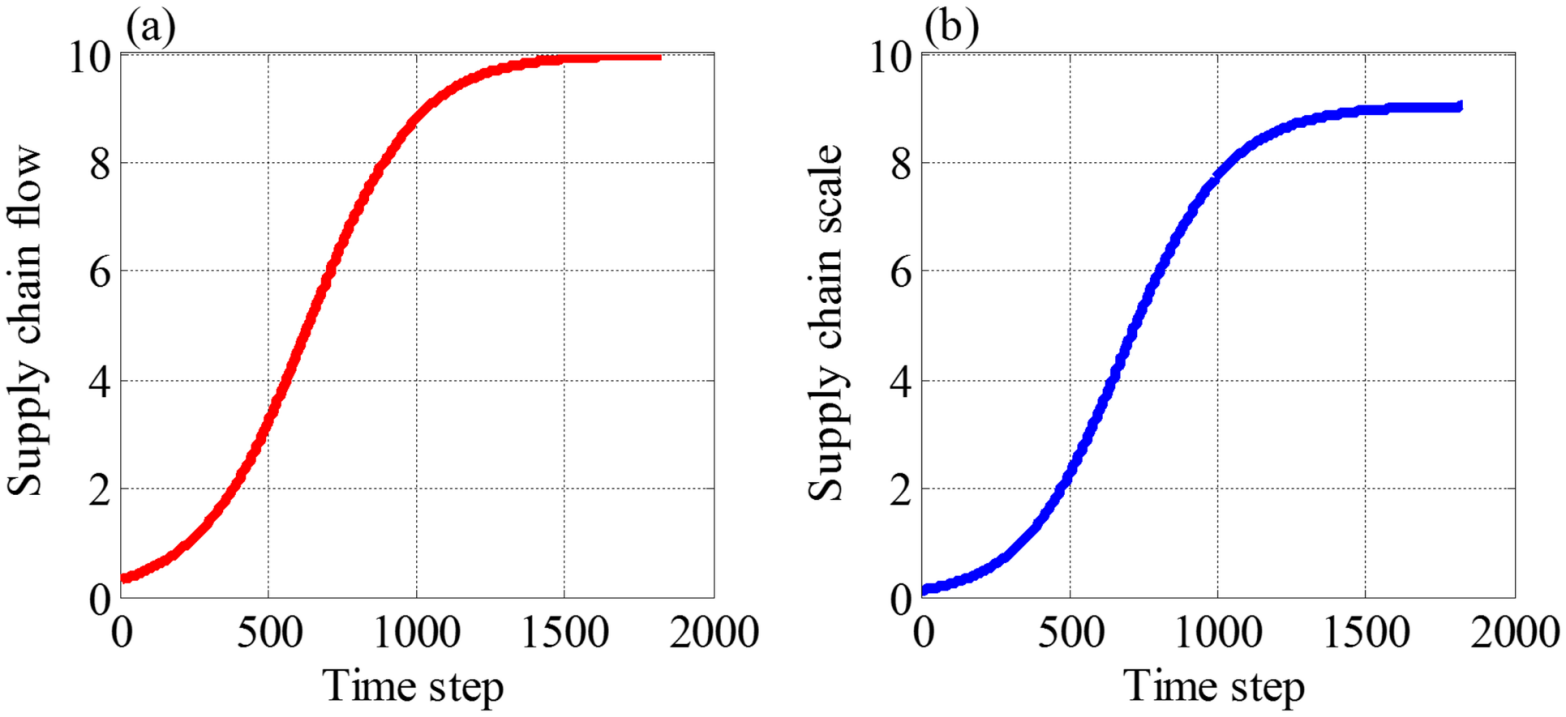
The schematic diagram about the change trends of flow and scale over time. The values are calculated according to Eqs (5) and (4). In these two equations, *F*_0_ = 0.3, *λ* = 0.818, *a* = 1, *b* = 0.1, *ε*_*F*_(*t*) is white noise, whose amplitude is 0.1.

Fig 2a and 2b show the trends of flow and scale respectively, which all satisfy Logistic curves and illustrate the development of supply chain has the feature of ecological growth.

**Rule 2: The change of enterprise competence follows two-stage mode**. Hirsch *et al*. divided the development of the enterprise competence into two stages as follows [45]:

**Stage 1** The growth rate is proportional to the enterprise competence level, while its capacity is weaker.

**Stage 2** The growth rate is inversely proportional to the enterprise competence level, while its competence is higher than average competence of the system.

Inspired by this work, we define the competence development of enterprise *v*_*i*_ as the following equation:

$$\frac{dc_{i}^{\left(n\right)}(t)}{dt}=\beta c_{i}^{\left(n\right)}(t)\left(1-\frac{c_{i}^{\left(n\right)}(t)}{\bar{c}}\right)+\varepsilon_{c}(t)$$ (6)

where $c_{i}^{\left(n\right)}(t)$ represents the competence of enterprise *v*_*i*_ at the time *t*, *n* presents node, *β* is natural growth rate of the enterprise, $\bar{c}$ denotes average competence of the members in the supply chain, and *ε*_*c*_(*t*) is also the white noise at the time *t*, and the amplitudes of it is fixed.

#### (2) The control of development stage

The development trend of supply chain should fulfill *Logistic curve* [44], which means that the increment ratio of the supply chain scale is first fast and then slow. We apply the probabilities of addition and deletion of nodes and edges to control the development phase of the whole supply chain network. Therefore, the probabilities of addition and deletion are closely associated with supply chain scale.

**Rule 3: Probabilities of addition and deletion is decided by supply chain scale**. Let *P*^(*A*)^(*t*) and *P*^(*D*)^(*t*) denote the probabilities of addition and deletion respectively, The addition includes node addition and edge addition, that is, *P*^(*A*)^(*t*) = *P*^(*NA*)^(*t*) + *P*^(*EA*)^(*t*), and so does the deletion. We define these probabilities according to the scale of supply chain as follows:

$$P^{\left(A\right)}(t)=\frac{1}{1+\text{exp}\left(\eta \left(1-\frac{M(t+1)}{M(t)}\right)\right)}$$ (7)

$$P^{\left(D\right)}(t)=1-P^{\left(A\right)}(t)=\frac{1}{1+\text{exp}\left(-\eta \left(1-\frac{M(t+1)}{M(t)}\right)\right)}$$ (8)

where, *η*(*η* > 0) is development factor of the scale, larger value of *η* indicates faster development speed of the supply chain; *M*(*t*) represents the supply chain scale at time *t*.

As *M*(*t* + 1) > *M*(*t*), the supply chain is in the phase of expansion, and we have *P*^(*A*)^(*t*) > 0.5 and *P*^(*A*)^(*t*) > *P*^(*D*)^(*t*); as *M*(*t* + 1) ≈ *M*(*t*), the supply chain is in the phase of stabilization, and *P*^(*A*)^(*t*) ≈ 0.5; as *M*(*t* + 1) < *M*(*t*), the supply chain is in the phase of decline and we have *P*^(*A*)^(*t*) < 0.5.

**Rule 4: Addition and deletion probabilities of node and edge**. We define the probabilities of node addition and edge addition as following:

$$P^{\left(NA\right)}(t)=\frac{P^{\left(A\right)}(t)}{1+\tau \text{exp}\left(\mu \left(0.5-P^{\left(A\right)}(t)\right)\right)}$$ (9)

$$P^{\left(EA\right)}(t)=P^{\left(A\right)}(t)-P^{\left(NA\right)}(t)=\frac{\tau P^{\left(A\right)}(t)}{\tau +\text{exp}\left(-\mu \left(0.5-P^{\left(A\right)}(t)\right)\right)}$$ (10)

where, *μ*(*μ* > 0) and *τ*(*τ* > 0) are the parameters in Eqs (9) and (10), which are increment factor and extreme factor of the supply chain, respectively. They represent decay rate and limit value about the probability of node addition.

From Eqs (9) and (10), the node addition plays a main role at the initial development phase, while the probabilities of node addition and edge addition finally tend to be stable, in consistence with the development of supply chain. As *τ* = 1, *P*^(*NA*)^(*t*) is finally equal to *P*^(*EA*)^(*t*); as *τ* < 1, *P*^(*NA*)^(*t*) is always greater than *P*^(*EA*)^(*t*); as *τ* > 1, *P*^(*EA*)^(*t*) will eventually exceed *P*^(*NA*)^(*t*).

Similarly, the probability of node deletion *P*^(*NA*)^(*t*) and the probability of edge deletion *P*^(*EA*)^(*t*) are represented as following:

$$P^{\left(ND\right)}(t)=\frac{P^{\left(D\right)}(t)}{1+\tau \text{exp}\left(\mu \left(0.5-P^{\left(D\right)}(t)\right)\right)}$$ (11)

$$P^{\left(ED\right)}(t)=P^{\left(D\right)}(t)-P^{\left(ND\right)}(t)=\frac{\tau P^{\left(D\right)}(t)}{\tau +\text{exp}\left(-\mu \left(0.5-P^{\left(D\right)}(t)\right)\right)}$$ (12)

In fact, the behaviors of mutual choice and elimination among the enterprises is also drawn on the ideas of agent-based approach, which can achieve flexible and autonomous activities for the design purpose. It has the features of autonomy, sociality, reactivity and initiative.

#### (3) Addition and deletion of node and edge in each layer

**Fig 3:**
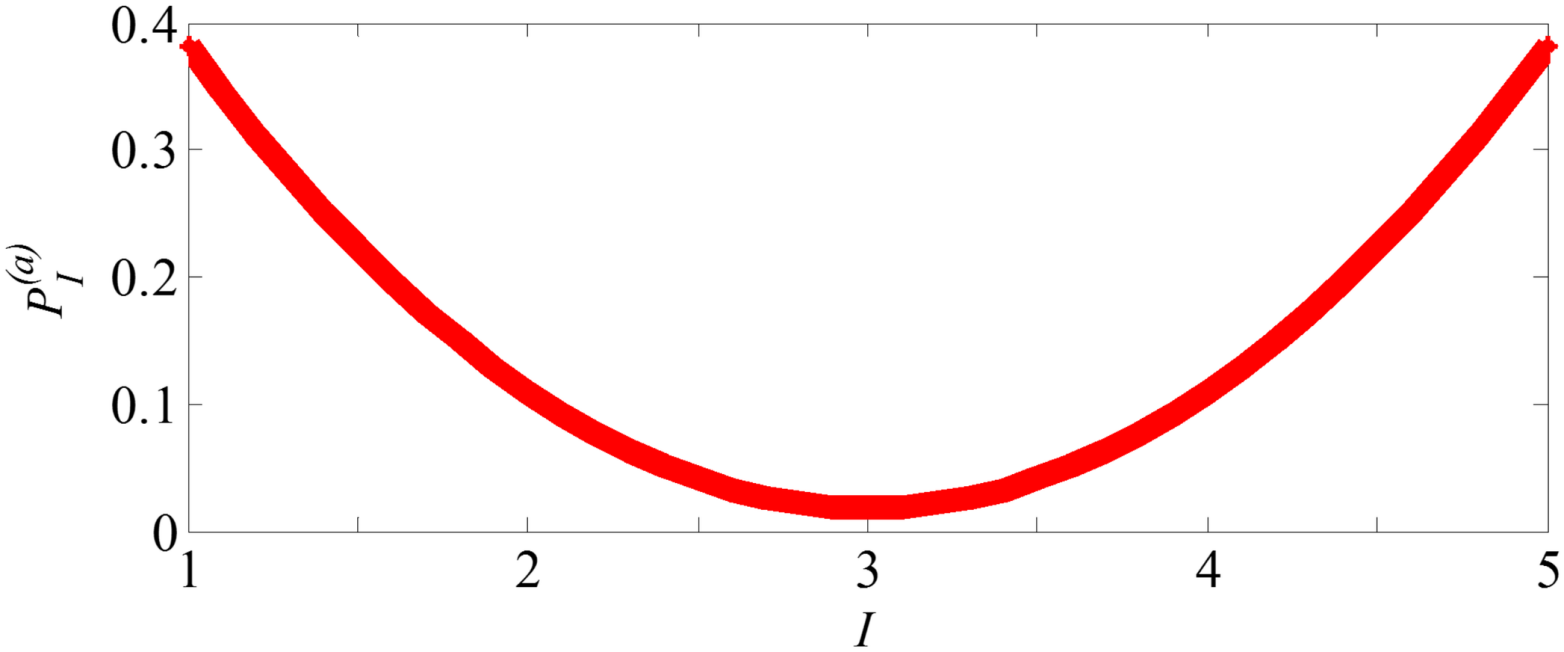
The layer active probability ($p_{I}^{\left(n\right)}$) as function of layer *I* is defined as smile curve.

**Rule 5: Layer active probability follows smile curve**. In the supply chain hierarchical system (as shown in Fig 1), core enterprises (manufactures) are relatively fixed, meanwhile the farther the enterprises is to the manufactures, the more active they can be. We use layer active probability to represent active degree of nodes in each layer. Thus the active probability $p_{I}^{\left(n\right)}$ in layer *I* can be defined as smile curve as shown in Fig 3 [46, 47]. Here $\sum_{I=1}^{L}p_{I}^{\left(n\right)}=1$. Hence, the probabilities of node addition and deletion in layer *I* at the time *t* are represented as follows:

$$p_{I}^{\left(na\right)}(t)=p_{I}^{\left(n\right)}P^{\left(NA\right)}(t)$$ (13)

$$p_{I}^{\left(nd\right)}(t)=p_{I}^{\left(n\right)}P^{\left(ND\right)}(t)$$ (14)

For a specific node *v*_*i*_ in the layer *I*, its deletion probability *p*^(*nd*)^(*v*_*i*_, *t*) can be calculated from layer active probability, node deletion probability and layer node weight as follows:

$$p_{}^{\left(nd\right)}(v_{i},t)=\frac{\text{exp}\left(-c_{i}^{\left(h\right)}(t)\right)}{\sum_{v_{k}\in {\tilde{H}}_{I}}\text{exp}\left(-c_{k}^{\left(h\right)}(t)\right)}p_{I}^{\left(n\right)}P^{\left(ND\right)}(t)$$ (15)

Meanwhile, the breakdown of partnership is more likely to happen between enterprises with lower corporation strength. Thus the deletion probability of edge (*v*_*i*_, *v*_*j*_) is associated with corresponding corporation strength, that is:

$$p_{}^{\left(ed\right)}((v_{i},v_{j}),t)=\frac{\text{exp}\left(-w_{ij}(t)\right)}{\sum_{(v_{k},v_{l})\in E}\text{exp}\left(-w_{kl}(t)\right)}P_{}^{\left(ED\right)}(t)$$ (16)

### Work flow of evolution model

**Fig 4:**
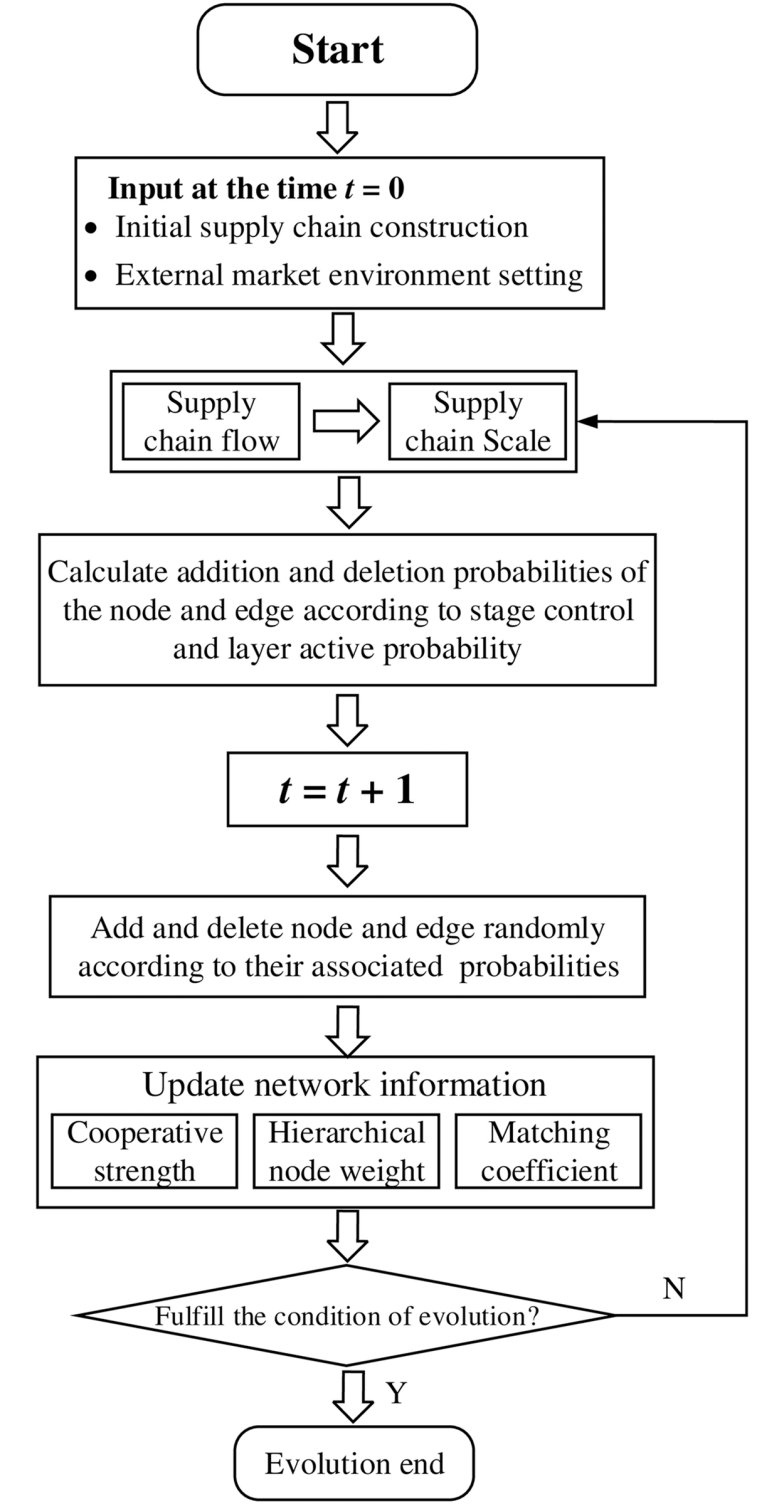
The flow diagram of the supply chain evolution.

Based on the above evolution rules, we build a new evolution model as shown in Fig 4. According to initial information of supply chain network and external market demand, we can calculate the flow and scale of the supply chain at time *t*, Then we obtain the addition and deletion probabilities of node and edge based on supply chain scale and layer active probability. When the evolution conditions are ready, we randomly add (or delete) one node (or one edge) in the giving supply chain network. Considering that the network balance will be broken after evolving at each time step, the network information need to be update. The evolution process will continually implement until satisfying termination condition.

### Detail illustration of evolution model

In this section, we illustrate the five steps of our evolution model in detail.

#### (1) Initialization of supply chain network and external market demand

At time *t* = 0, we randomly construct a small hierarchical network as initial supply chain network and set initial information of this network, including enterprise competences and cooperative strengths among the enterprises. We also assign layer-layer association probabilities for the network in construction. Meanwhile, under the assumption that the external market demand is relatively stable, we define parameters that describe the external market demand, including the nature growth rate, the retardation coefficient of the flow, and random disturbance.

#### (2) Calculation of supply chain flow and scale

The supply chain scale and flow at the time *t* can be calculated by Eqs (5) and (6) using parameters of external market demand.

#### (3) Calculation of the addition and deletion probabilities of node and edge

Based on the supply chain scale, the addition and deletion probabilities of node and edge in the whole supply chain are obtained by Eqs (7)–(10).

#### (4) Stochastic evolution of supply chain network

We change the topology of the supply chain network at time *t* to obtain network at time *t*+1. This is done through randomly adding and deleting nodes and edges according to the probabilities of node and edge addition and deletion in each layer. The detail process is as follows:

**Node addition** To add a new node, we first randomly determine the layer *I* through addition probability $p_{I}^{\left(na\right)}(t)$ of layer node. Then we chose another layer *J* in which some nodes may connect with the new node according to layer-layer association probability $p_{IJ}^{\left(h\right)}$. The partners of the new node are chosen by matching coefficients *z*_*ij*_(*t*). When new node *v*_*i*_ in layer *I* corporates with its partner *v*_*j*_ in layer *J*, the corporation strength (weight) between them should be proportional to matching coefficient and average value of corporation strengths of the two layers. Then we calculate the weight of the partnership between the new node *v*_*i*_ and existed node *v*_*j*_ at time *t* + 1 as follows:

$$w_{ij}^{\left(0\right)}(t+1)=z_{ij}(t)\times \frac{q_{IJ}^{\left(h\right)}(t)}{\left\|\left\{(v_{k},v_{l})\in E|v_{k}\in {\tilde{H}}_{I}\&v_{l}\in {\tilde{H}}_{J}\right\}\right\|}$$ (17)

**Node deletion** We first calculate the node deletion probability of each node by Eq (15). Then we delete a node randomly according to this probability. Once a node is deleted, all of its links will also be cut off.

**Edge addition** To add a new partnership, the layer pair of the new partnership is chosen randomly by layer-layer association probability $p_{IJ}^{\left(h\right)}$. Then the new partnership between two nodes which have not cooperated before is selected randomly through matching coefficient. At last, we calculate the weight of this new link by Eq (17).

**Edge deletion** The partnership is deleted randomly according to deletion probability of edge calculated by Eq (16).

It is worth to note that the deletion may cause the separation of local nodes and edges from the whole network. However, since the deleted enterprise or partnership has weaker competence or cooperative strength, the lost local nodes and edges do not have big influence to the whole supply chain network.

#### (5) Information update of supply chain network

Both the node and edge addition (deletion) will affect the balance of supply chain. However, the demand of stable development and self-interest seeking of the enterprises will drive the supply chain to rebalance as soon as possible. The balance of supply chain includes the stabilization of cooperation strength among the layers, the balances between the input strengths and output strengths of enterprises, and the assignment stabilization of the market share among the enterprises in the same layer. In this work, the new balance is based on the former balance state. The adjustment of new balance starts from the manufacturer enterprises and implements toward the upstream and downstream of the manufacturers.

#### The update of output strength for the manufacturers

At time *t* + 1, we first calculate temporary cooperative strength of a new edge by Eq (17), while we set the temporary weights of other edges as their weights at time *t*, *i*.*e*., $w_{ij}^{\left(0\right)}(t+1)=w_{ij}(t)$. We then calculate the total output flow *F*(*t* + 1) of the manufacturer at time *t* + 1 by Eq (5). At last, for each manufacturer *v*_*i*_ in the manufacturer layer *I*, its output strength to enterprise *v*_*j*_ (weight of edge (*v*_*i*_, *v*_*j*_)) is obtained as following:

$$w_{ij}^{}(t+1)=\frac{w_{ij}^{\left(0\right)}(t+1)}{\sum_{v_{k}\in {\tilde{H}}_{I}\&(v_{k},v_{l})\in E}w_{kl}^{\left(0\right)}(t+1)}F(t+1)$$ (18)

#### The update of output strengths for downstream enterprises of manufactures

It starts with output edges of the manufacturers and updates toward the downstream of manufacturers layer by layer. For node *v*_*i*_, its output strength (weight of edge (*v*_*i*_, *v*_*j*_)) is decided by its input strength, temporary cooperative strength and matching coefficient, which is calculated as follows:

$$w_{ij}(t+1)=\left(1+\rho_{i}^{\left(0\right)}\right)\sum_{(v_{k},v_{i})\in E}w_{ki}^{\left(0\right)}(t+1)\times \left(\alpha_{w}\frac{w_{ij}^{\left(0\right)}(t+1)}{\sum_{(v_{i},v_{k})\in E}w_{ik}^{\left(0\right)}(t+1)}+\left(1-\alpha_{w}\right)\frac{z_{ij}^{}(t)}{\sum_{(v_{i},v_{k})\in E}z_{ik}^{}(t)}\right)$$ (19)

where, *α*_*w*_ is a parameter that adjusts the contribution of temporary cooperative strength and matching coefficient. Considering the stability of cooperative strength, it should satisfy 0.5 ≪ *α*_*w*_ < 1. $\rho_{i}^{\left(0\right)}$ is growth rate of the *v*_*j*_’s value in the supply chain. Since each enterprise can create value, when the materials flow through it, the output strength of the enterprise *v*_*i*_ is $\left(1+\rho_{j}^{\left(0\right)}\right)$ times greater than the input strength.

#### The update of input strength for the manufacturers and their upstream enterprises

This update starts from the input edges of the manufacturers, and implements toward the upstream of manufacturers layer by layer. The input strength of node *v*_*j*_ is updated as follows:

$$w_{ij}(t+1)=\frac{\sum_{(v_{j},v_{k})\in E}w_{jk}^{}(t+1)}{1+\rho_{j}^{\left(0\right)}}\times \left(\alpha_{w}\frac{w_{ij}^{\left(0\right)}(t+1)}{\sum_{(v_{k},v_{j})\in E}w_{kj}^{\left(0\right)}(t+1)}+\left(1-\alpha_{w}\right)\frac{z_{ij}^{}(t)}{\sum_{(v_{k},v_{j})\in E}z_{kj}^{}(t)}\right)$$ (20)

Where, *α*_*w*_ and $\rho_{j}^{\left(0\right)}$ have same meanings as in Eq (19).

#### The update of layer node weight

When the update for all cooperation strengths finishes, we can update the layer node weights. We first calculate enterprise competence $c_{i}^{\left(n\right)}(t+1)$ by Eq (6). Then the layer node weight of *v*_*i*_ in layer *I* is updated as follows:

$$c_{i}^{\left(h\right)}(t+1)=\alpha_{c}\frac{c_{i}^{\left(n\right)}(t+1)}{\sum_{v_{j}\in {\tilde{H}}_{I}}c_{j}^{\left(n\right)}(t+1)}+(1-\alpha_{c})\frac{\sum_{\left(v_{i},v_{j}\right)\in E}w_{ij}(t+1)}{\sum_{v_{k}\in {\tilde{H}}_{I}\&\left(v_{k},v_{j}\right)\in E}w_{kj}(t+1)}$$ (21)

where, *α*_*c*_ is a parameter that adjusts the contribution of enterprise competence and cooperative strength.

#### The update of matching coefficient

According to Eq (3), the matching coefficient between two nodes can be updated, and then *z*_*ij*_(*t* + 1) is got.

### Network topology indices

This work applies four indices to measure the properties of the network evolved by our model, which are node number, edge number, average layer link density and average strength.

#### (1) Node number, edge number

In the supply chain, the node represents enterprise, and the edge represents the partnership between the enterprises. The change trends of node number and edge number could appropriately describe the scale of supply chain in its evolution process.

#### (2) Average layer link density

Different from common networks, two nodes in the same layer of supply chain network do not build partnership. In order to describe the closeness among the layers, we propose the concept of layer link density. The layer link density *LD*_*i*_ of node *v*_*i*_ is defined as following:

$$LD_{i}=M_{i}/M_{i}^{\text{max}}$$ (22)

where *M*_*i*_ is the number of edges connecting to node *v*_*i*_, $M_{i}{}^{\text{max}}$ denotes maximum number of edges that may link to node *v*_*i*_. The average layer link density *ALD*_*I*_ of layer *I* is the average value of layer link density for all nodes in layer *I*. The average layer link density of the whole network is similarly defined.

#### (3) Average strength

Average strength of the network is the average value of all nodes’ strength in the network. It reflects the average contribution degree of node enterprise to the supply chain. The average strength of each layer is similarly defined.

## Results and discussion

In this section, we conduct some simulations to verify our evolution model for the supply chain. The discussion mainly includes the analysis of evolutions in various time steps, effects of external market demand and internal competition-cooperation to network evolutions, and benchmark data validation.

**Fig 5:**
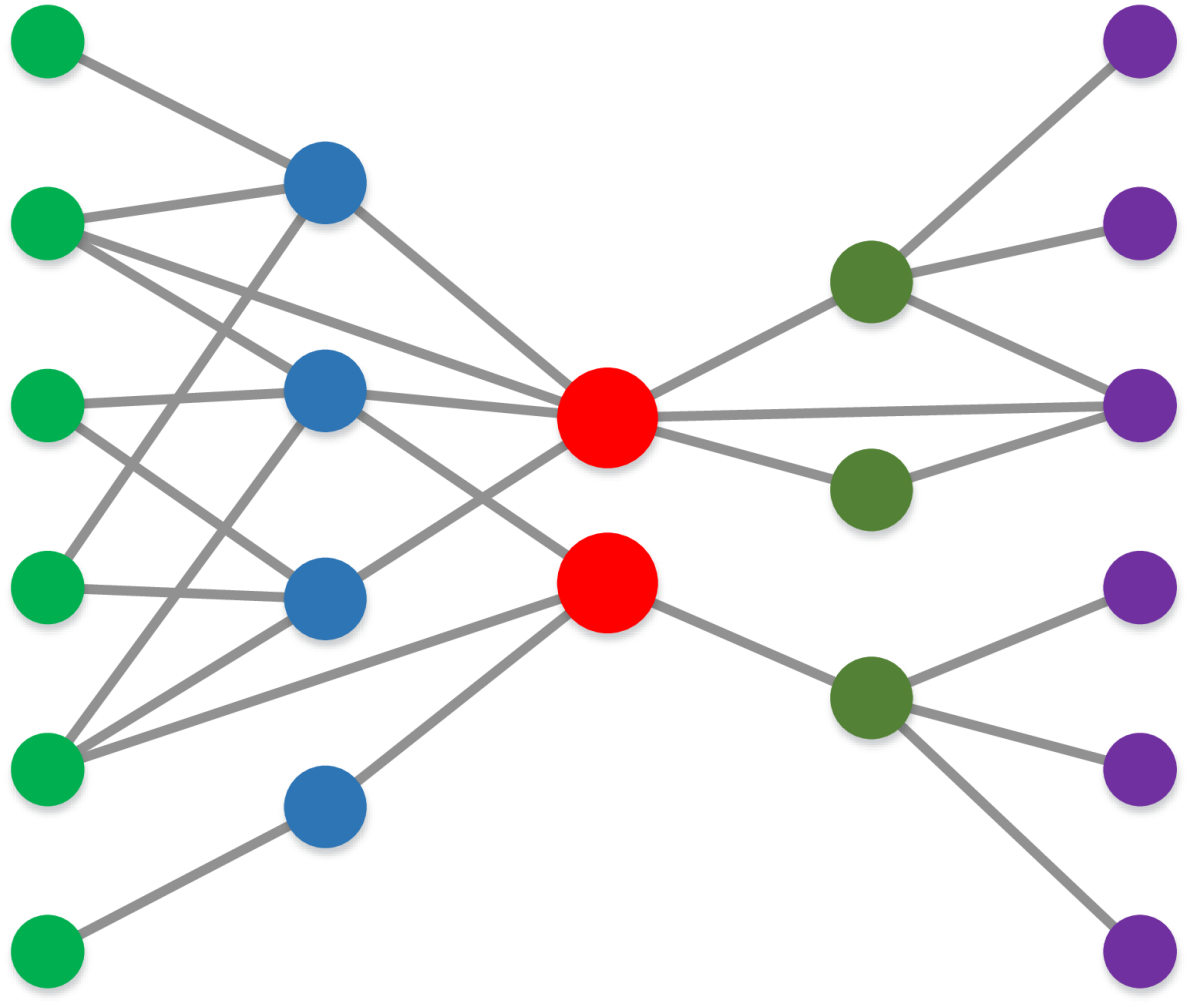
The initial supply chain network we select, which includes five layers, 21 node enterprises, 28 partnerships (edges) and two manufactures. The 3rd layer is manufacture layer.

In simulation section, we take the supply chain with five layers as an example, because it is a type of classic supply chain, in which, five layers present raw material purchasing, semi-finished products processing, manufacturing, warehousing (distribution), and retail processing. As shown in Fig 5, the initial supply chain network we select is a hierarchical network with 2 core manufacturers, which includes 5 layers, 21 enterprises (nodes) and 28 partnerships (edges). The 3rd layer is manufacture layer. The initial information of enterprise competences, cooperative strengths among the enterprises and layer-layer association probabilities are set randomly.

To minimize the impact of randomness, all the simulations are repeated 30 times and then the average values of the 30 simulation results are used for further analysis. Considering the features of supply chain with manufactures as the core, this work applies four indices, *i*.*e*., node number, edge number, average layer link density and average strength, to measure the property of the network structure.

### Analysis of networks constructed by evolution model at different time step

Since the supply chain develops continually with the time, it is necessary to analyze the development trend through simulating the evolution model and discuss the network properties in different time steps. We will analyze the node numbers, the edge numbers, average layer link densities and average strengths of the whole network and each layer, along with the changing of time step *t*, respectively.

The simulation is implemented based on the initial network shown in Fig 5. In this section, in order to ensure the development of supply chain does not decline, we set the initial values of parameters for the evolution model as follows:

$$a=1,\;b=0.1,\;\beta =0.05,\;\eta =500,\;\mu =10,\;\tau =2,\;\alpha_{c}=0.5,\;\alpha_{w}=0.9.$$

**Fig 6:**
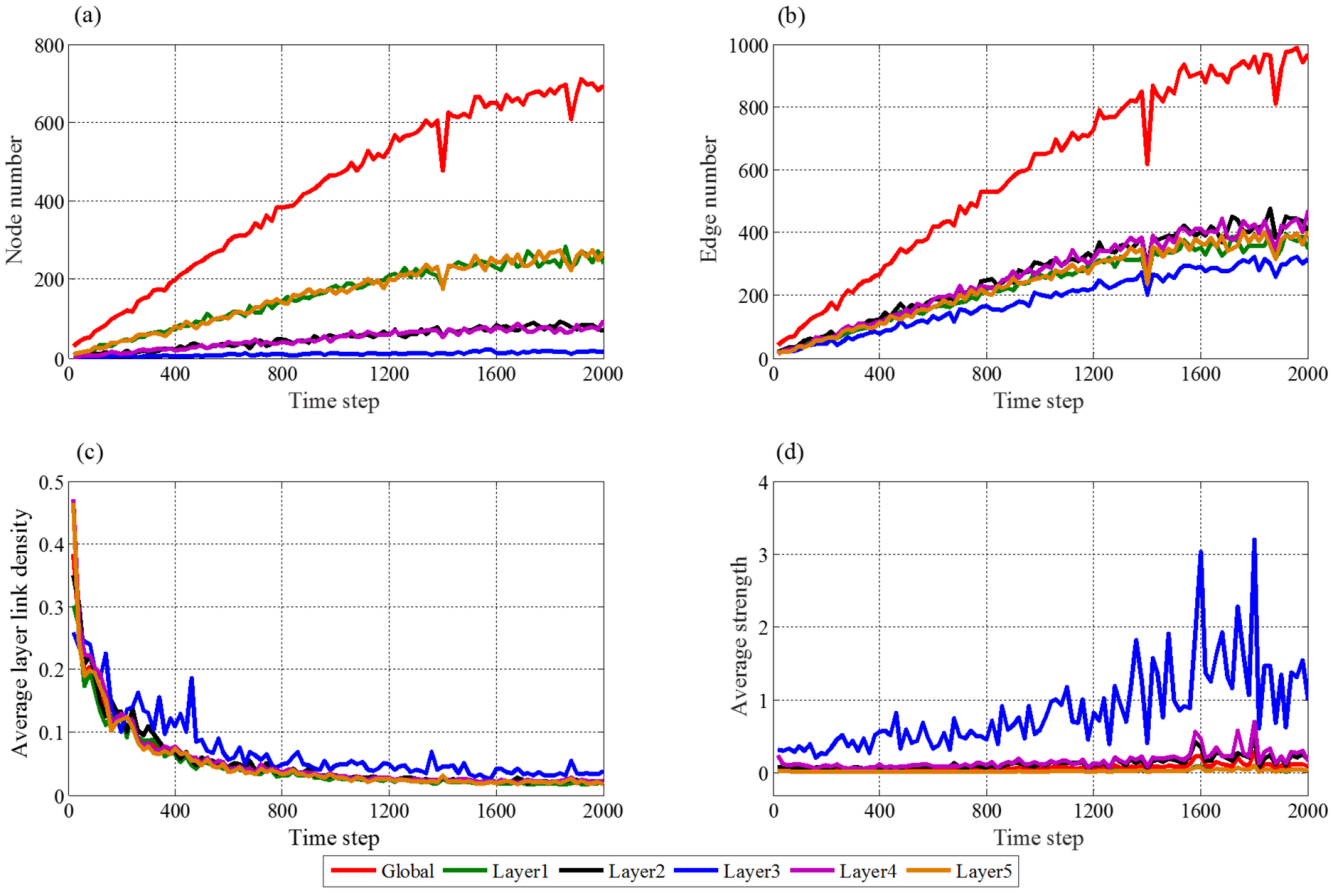
The trends about four indices of the whole supply chain and five layers with the change of time step, where (a) is the trends of node number, (b) is the trends of edge number, (c) is the trends of average layer link density, (d)is the trends of average strength.

*ε*_*F*_(*t*) is white noise, whose amplitude is 0.1. *t* changes from 0 to 2000 with the step of 20. Then the simulation is conducted, the networks are constructed and four indices are obtained, which are shown in Fig 6.

Fig 6(a) shows that, the increment rates of all the six curves of node numbers get slower and slower over time, at last they are close to zero. These trends satisfy the development law of the supply chain. At earlier stages of the supply chain development, node addition plays main role, then the probability of node addition is gradually close to that of the node deletion, when the supply chain gets mature. Additionally, the growths of nodes in the first and fifth layers are fastest, those in the second and fourth layers come second, while that in the third layer is slowest. This means that the farther the layer to the manufacture layer, the faster the increasing rate of nodes, which illustrates that the manufactures have dominant position in the supply chain, and it is hard to add enterprises that compete with existing manufactures. Fig 6(b) shows development trend of the partnerships (edges) among the enterprises. Similarly, all the six curves increase with time, while the growth rates of them decrease constantly. The main reason is that, the development speed of supply chain is first fast and then slow, in which, the node and edge addition play the main role in the early stage. Then deletion probabilities of the node and edge gradually increase and addition probabilities gradually decrease with the development of supply chain. Finally, addition probabilities are close to deletion probabilities.

In Fig 6(c), six curves present decreasing trends. According to Eq (22), the average layer link density shows the closeness degree of node with layers of its upstream and downstream. With the development of the supply chain, its node scale becomes larger and larger, and the ratio of nodes’ partners to all possible links between nodes is lower and lower. Thus all average layer link densities become smaller and smaller.

Fig 6(d) gives the trends of average strengths in the development of supply chain network. Average strengths in the whole network and in each layer show slight increasing trends. It indicates that the businesses and profits of the enterprises in each layer increase with the development of the supply chain, until they reach balance. Since manufactures are core of supply chain studied in this work, they play dominant role in the development process of supply chain. Thus the average strength of the third layer is far greater than that of other four layers.

From above analysis about the four indices for networks constructed by our evolution model, we can see that the evolution of our model, which is based on the five rules described in the Methods section, is consistent with real development process of supply chain. Meanwhile, the constructed networks have the characteristics of real supply chains with manufactures as the core. Additionally, the change trends of indices with time step in five layers are remarkably similar with that in the whole network, thus, we only analyze the effects of external market demand and internal competition-cooperation to whole network in the following two sections.

### Effects of external market demand to the evolution model

The scale of the supply chain is decided by its flow. According to Eq (6), the flow is closely associated with external market demand, which is mainly described by three parameters, *i*.*e*., nature growth rate *a*, retardation coefficient *b* and the amplitude of random disturbance *ε*_*F*_(*t*). Once these parameters change, the flow and scale of the whole supply chain will be affected, so will be the evolution process. We use these three parameters to depict external market demand.

To investigate the impacts of external market demand to the evolution model, we perform three groups of simulation experiments. In each group of experiment, we let one of the three parameters change in a specific area with a fixed step and take the other two parameters as their initial values as in last section. We simulate our evolution model with 1000 time steps and obtain the evolved networks. Then we study topological features of these networks. The change area and step of each parameter is listed in Table 1.

**Table 1:** The change ranges of the three parameters about external market demand.

Parameter	Interval	Step
*a*	[0, 25]	0.1
*b*	[0, 2.4]	0.01
Amplitude of white noise *ε*_*F*_(*t*)	[0, 1]	0.01

#### (1) Nature growth rate

**Fig 7:**
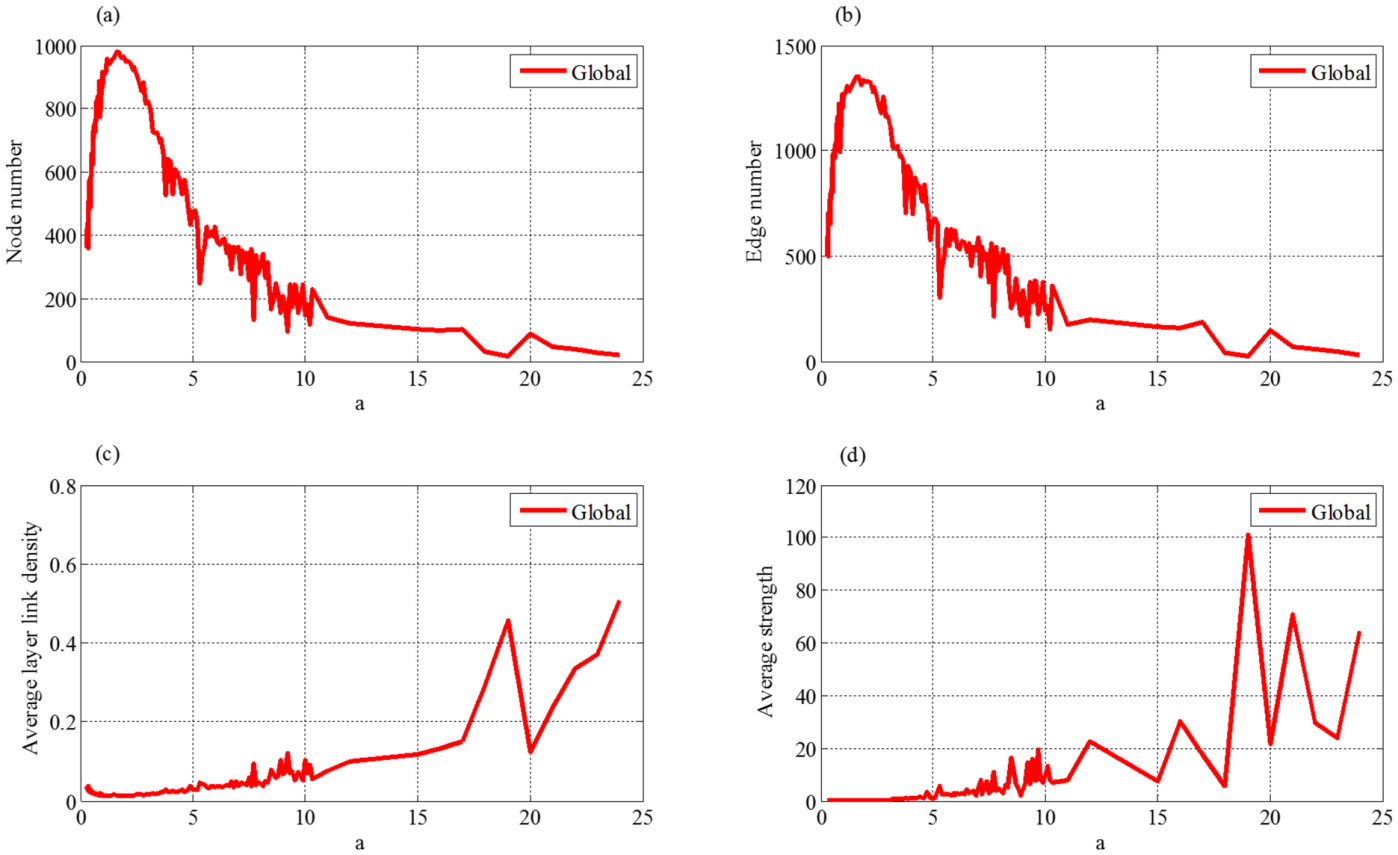
Effect of nature growth rate to four indices of evolution network, where (a) is node number, (b) is edge number, (c) is average layer link density and (d) is average strength.

As Fig 7(a) and 7(b) shows, when *a* just begins to increase, the numbers of nodes and edges increase. Specifically, when *a* = 2, the numbers of nodes and edges reach the maximum value. Actually, the increasing of nature growth rate *a* leads to the increment of supply chain flow and the expansion of supply chain scale. Thus both the numbers of cooperative enterprises and partnerships increase during this period. However, as the parameter *a* is greater than a certain value, the numbers of nodes and edges first decrease and then come to stability. This means that when the growth rate increases, the supply chain flow will exceed market demand and become saturated, thus the development of supply chain is hindered. Excessive expansion of the supply chain will cause internal competition become white hot and the development of the supply chain is lopsided. At this stage, weak enterprises and partnerships will be eliminated from the supply chain, and then the supply chain network is mainly constructed by the remaining enterprises and their partnerships with stronger competences or strengths. For example, in the previous years, with the emerging of mobile phone industry, numerous manufacturers expanded their capacity for more interest. However, this blind production resulted in market saturation of mobile phone industry. Eventually, a large number of mobile phone manufacturers went bankrupt in the fierce competition.

Fig 7(c) and 7(d) shows that the average layer link density and average strength keep stable, when the parameter *a* of external market demand changes little. These results suggest that network structure of supply chain is affected slightly as *a* changes in a reasonable range. However, these two indices start to increase with further increment of the parameter *a*. According to the analysis of the node number and edge number, their sizes will decrease as *a* increases. Then the remaining connections among the enterprises will become relatively denser. Thus the average layer link density will increase. Meanwhile, the contribution of each enterprise (average strength) to the whole supply chain will enhance, because the network constructed by the rest enterprises and partnerships are with stronger competences and strengths.

#### (2) Retardation coefficient

**Fig 8:**
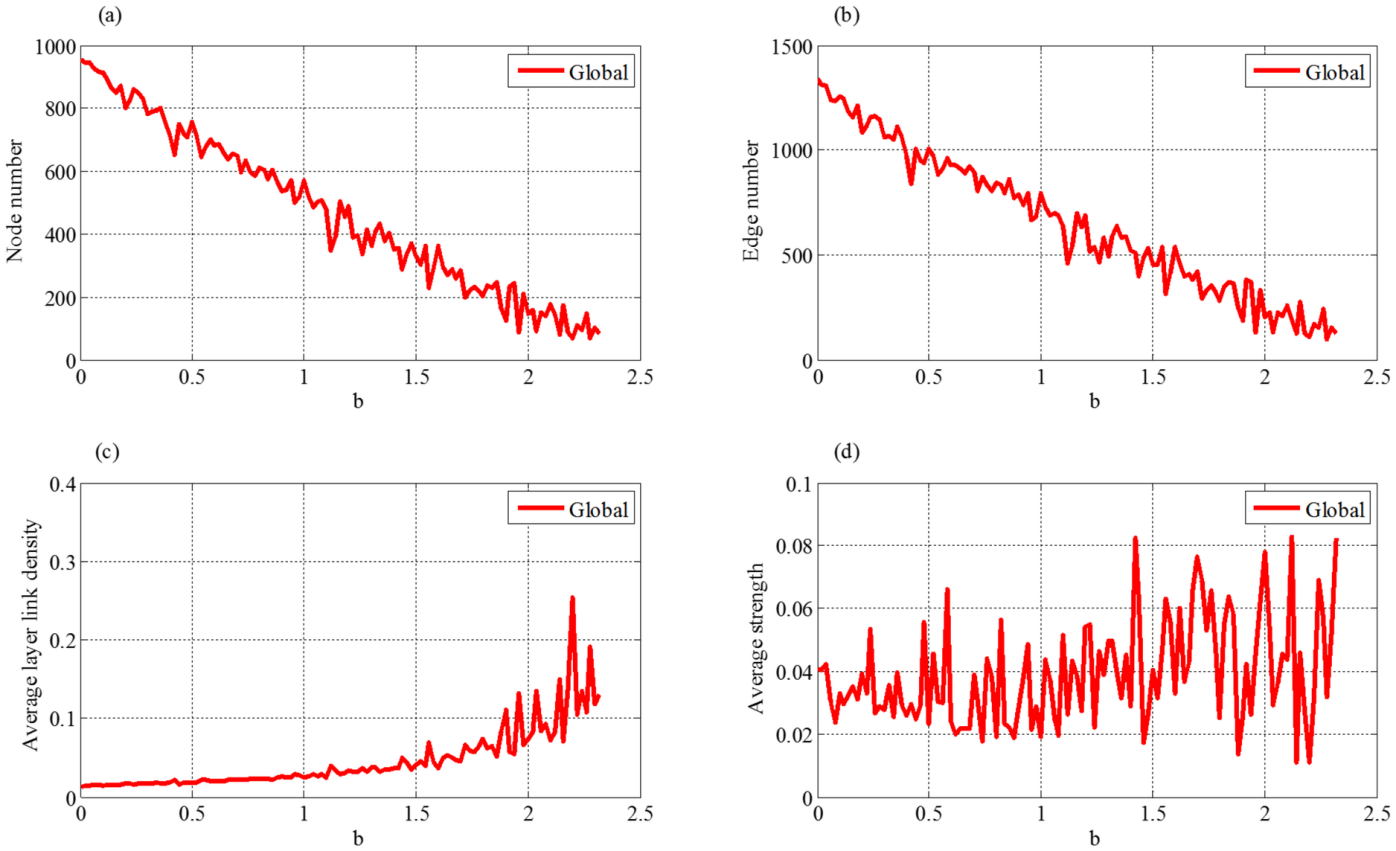
Effect of retardation coefficient to four indices of evolution network, where (a) is node number, (b) is edge number, (c) is average layer link density and (d) is average strength.

In Fig 8(a) and 8(b), both node and edge numbers monotonously decrease with the increasing of retardation coefficient *b*. These results suggest that the retardation of external market demand will decrease the growth rate of the supply chain and then inhibit the scale development of the supply chain, which will lead to stronger competition among the enterprises. Hence, only the enterprises and their partnerships with stronger competences and strengths can be reserved.

Fig 8(c) shows that the average layer link density first keeps stable and then increases with retardation coefficient *b*. The stable stage illustrates that the supply chain has the ability to resist outside interference in the premise of smaller retardation. When parameter *b* exceeds a certain value, the remaining enterprises will build more connections with each other, exhibiting a trend of local alliance. From Fig 8(d), we can see that the average strength is not sensitive to the change of retardation coefficient. However, it shows a trend of greater and greater fluctuation with the increasing of *b*, because the competition becomes more and more fierce.

#### (3) Random disturbance

**Fig 9:**
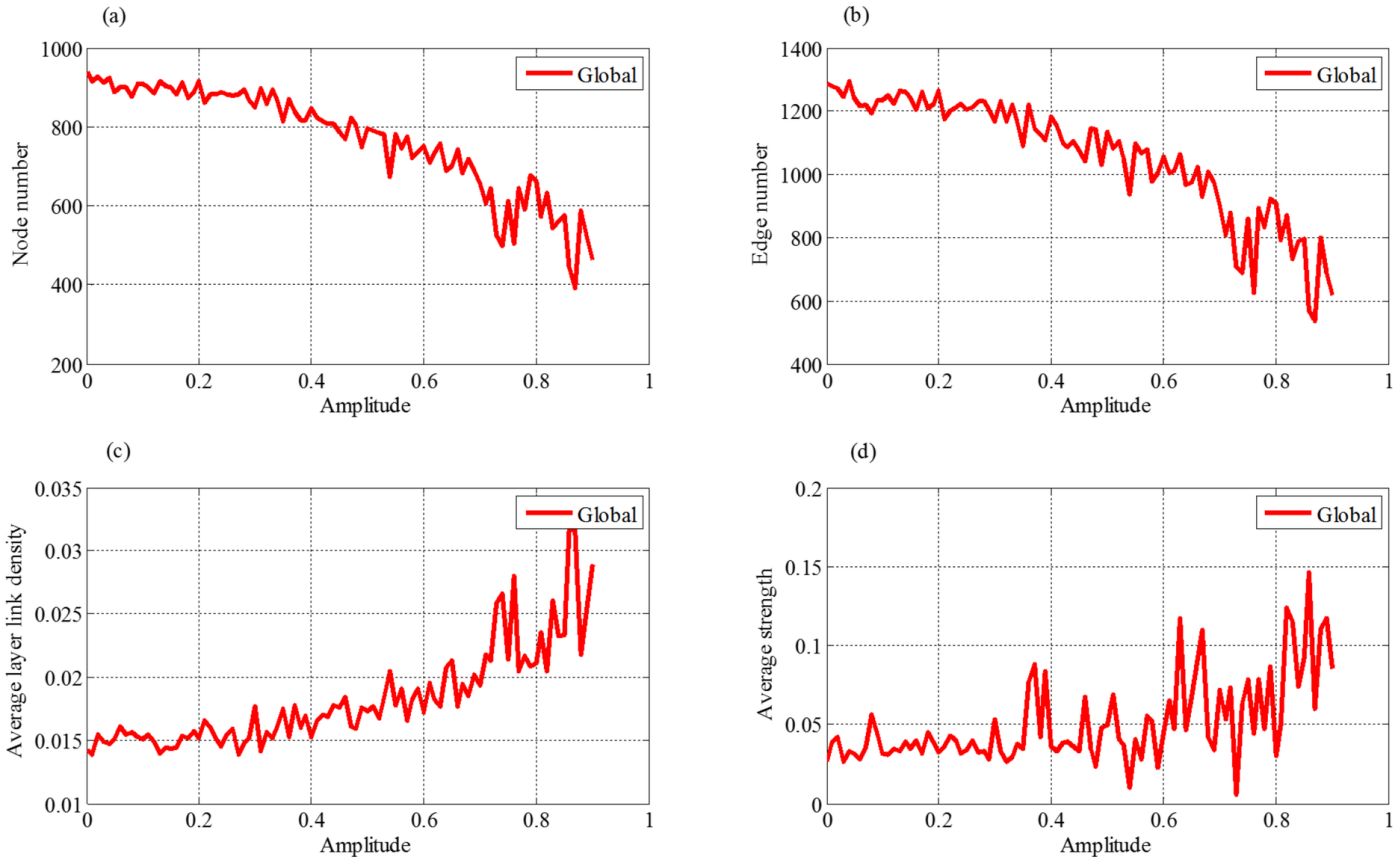
Effect of random disturbance to four indices of evolution network, where (a) is node number, (b) is edge number, (c) is average layer link density and (d) is average strength.

From Fig 9(a) and 9(b), we can see that, when the amplitude of white noise *ε*_*F*_(*t*) does not exceed 0.4, both the node number and the edge number fluctuate at certain intervals. However, as the amplitude further increases, they show decreasing trends. Especially, the network will breakdown, as the amplitude exceeds 0.9. These results suggest that the development of the supply chain can withstand limited external interference, but it will enter recession stage if the external uncertainty and interference are beyond its tolerance range.

In Fig 9(c) and 9(d), as the change of amplitude does not exceed 0.4, the average layer link density and average strength will fluctuate in certain range, suggesting the topological structure of the network changes little. It indirectly explains that the supply chain has some anti-interference ability. When amplitude exceeds 0.4, the size of the network will become small, and then the node number and edge number will decrease. Hence, the connection among the remaining enterprises becomes relatively denser. At the same time, the average strength shows a slightly increasing and fluctuation trend. However, when the amplitude becomes large enough (larger than 0.9 in Fig 9(c) and 9(d)), we cannot obtain connected supply chain networks. This means that the supply chain will collapse if external uncertainty and interference exceed sustainable range.

Therefore, during the process of supply chain development, the speed of nature growth should be controlled in a reasonable range, which can promote the health development of supply chain. Too slow and too fast speed will cause negative effect to supply chain development. Based on the above results about retardation coefficient and random disturbance, we can find that supply chain system has a certain ability to adapt the external market demand. However, the supply chain’s ability of withstanding external interference is limited. Thus, the managers of supply chain should conduct risk management and minimize the influence of external interference to supply chain.

### Effects of internal competition-cooperation to the evolution model

Besides the external market demand, the changes of internal competition and cooperation also affect the evolution of the supply chain. The competition-cooperation mechanisms mainly include supply chain development trend, enterprise’s entry and exit, partnership’s establishment and break, and the change of enterprise’s competence. These factors are described by the scale development factor *η*, extreme factor *τ* and increment factor *u*, nature growth rate *β* of enterprise competence, and weight *α*_*c*_ and *α*_*w*_. Our model uses six parameters to depict internal competition and cooperation mechanisms. To illustrate the effects of internal competition and cooperation mechanisms to the evolution model, we conduct similar method as that of external market demand. The change areas and steps of six parameters are shown in Table 2.

**Table 2:** The change ranges of the six parameters about internal competition and cooperation mechanisms.

Parameter	Interval	Step
*η*	[0, 3000]	100
*τ*	[0, 100]	1
*u*	[0, 100]	10
*β*	[1, 3]	0.01
*α*_*c*_	[0.5, 1]	0.01
*α*_*w*_	[0, 1]	0.01

**Fig 10:**
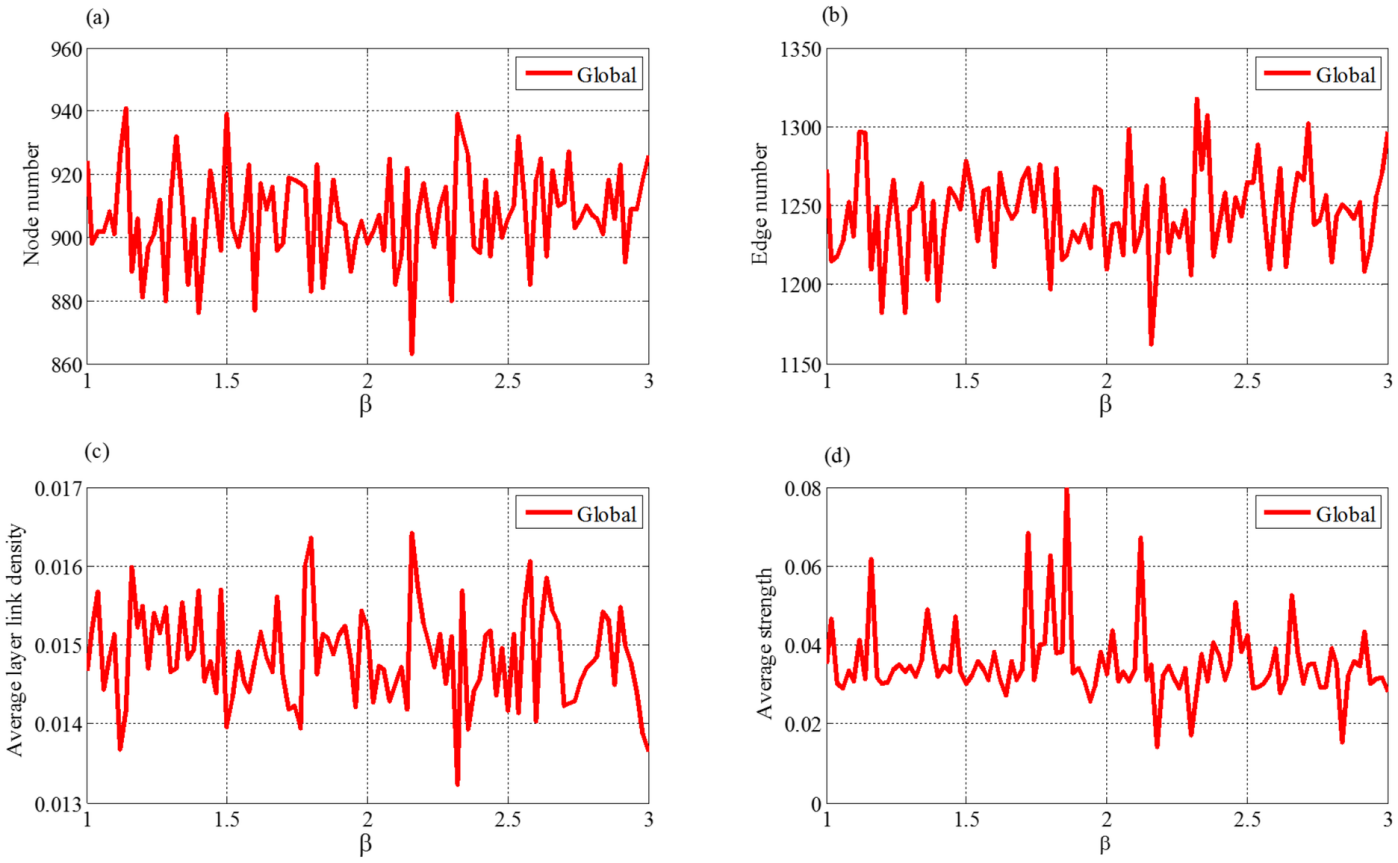
The trends of four topological indices of evolving supply chain network with the change of *β*. (a) node number, (b) edge number, (c) average layer link density, (d) average strength.

As shown in Fig 10, Figure A and Figure B in S1 File, our simulations suggest that the increases of parameters *β*, *α*_*c*_ and *α*_*w*_ do not significantly affect node number, edge number, average layer link density and average strength of the networks. That is to say, all these four topological indices are not sensitive to the three parameters. The reason may be that these three parameters are mainly associated with the evolutions of the enterprise competence and the partnership strength. Their effects to the topological features of the whole supply chain network are minuscule. In the following, we focus on discussing the other three parameters.

#### (1) Scale development factor *η* and increment factor *μ*

**Fig 11:**
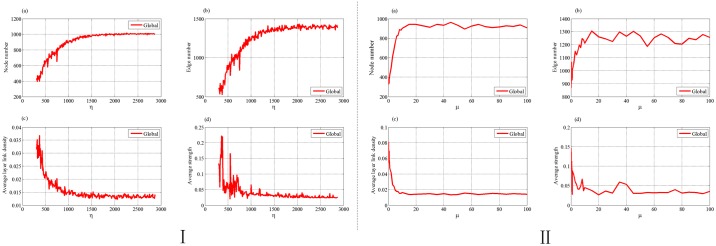
The trends of four topological indices of evolved supply chain network with the change of *η* (I) and *μ*(II). (a) node number, (b) edge number, (c) average layer link density, (d) average strength.

Fig 11I(a-b) and 11II(a-b) show that the numbers of nodes and edges increase with the increment of *η* and *μ*. As *η* and *μ* reach a certain value, they change little and reach stable status. Because all the networks are constructed by simulating our evolution model for 1000 steps, this suggests that networks constructed by the model with smaller *η* and *μ* reach saturation slower than those by model with larger *η* and *μ*, which is in consistence with the development of the scale of supply chain.

In Fig 11I(c-d) and 11II(c-d), with the increasing of *η* and *μ*, average layer link densities and average strengths first significantly decrease, and then reach a relatively stable status. *η* is scale development factor. With the increasing of *η* and *μ*, the growths of the supply chain will accelerate. Then more enterprises enter the supply chain, making the network sparser. Therefore, the average layer link density and average strength decrease with the increasing of *η* and *μ*. The smaller the value of *η*, the greater the deletion probability and the more likely that important enterprises are deleted, then the greater the fluctuation of average strength.

#### (2) Extreme factor *τ*

**Fig 12:**
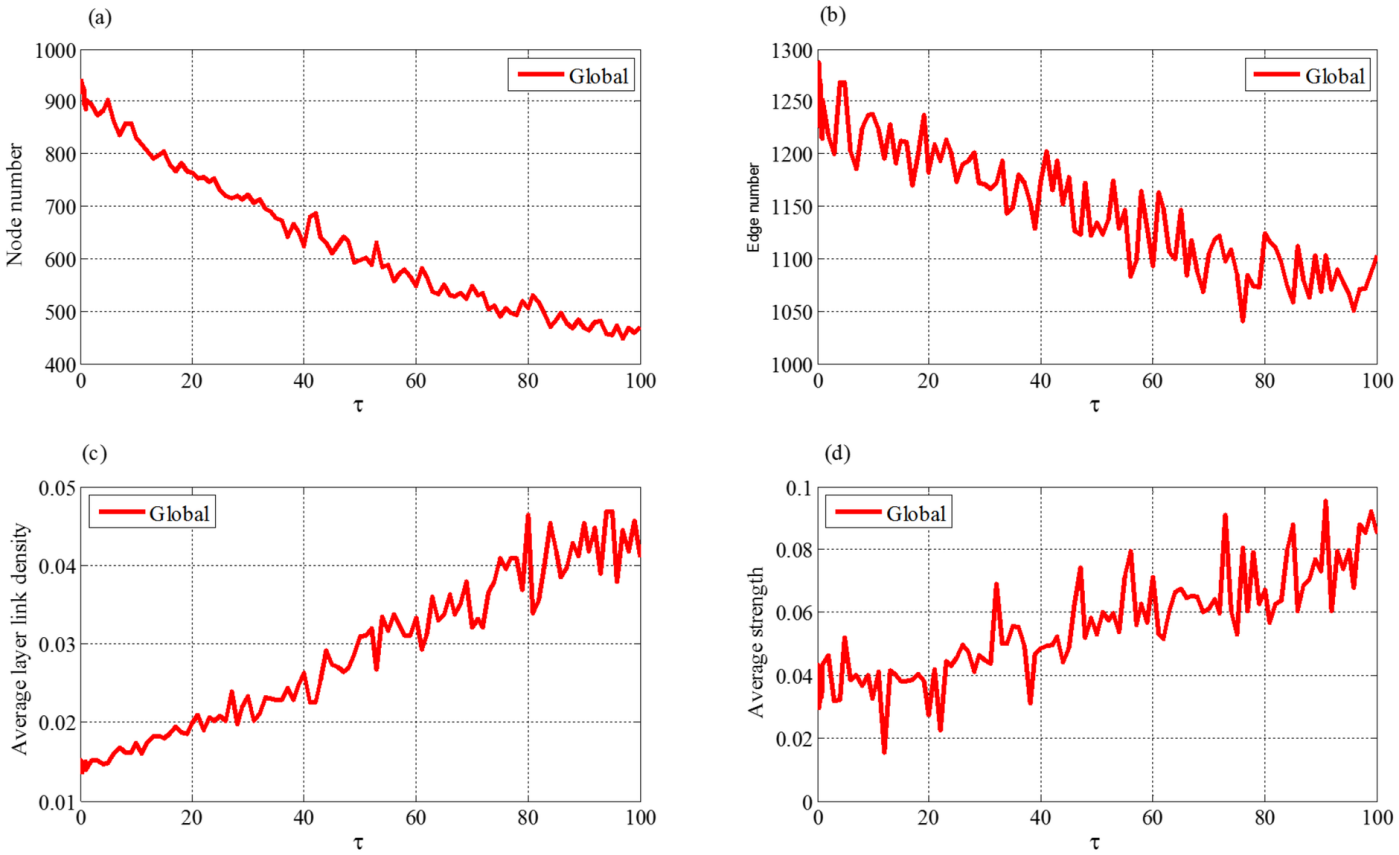
The trends of four topological indices of evolved supply chain network with the change of *τ*. (a) node number, (b) edge number, (c) average layer link density, (d) average strength.

Fig 12(a) shows that, when *τ* increases, both the numbers of nodes and edges will decrease first and then get stable status. From Eqs (9) and (10) we can see that, the increment of *τ* leads to the decreasing of the node addition probability. Thus it is difficult for the network to further expand and hardly reach the saturation status. At one step, conducting one node addition leads to the adding of at least one more edge to the network, whereas conducting one edge addition only lets the network get one new edge. Thus the increasing of edge number caused by node addition is greater than or equal to that of edge addition. Therefore, when the probability of node addition becomes small, the number of the edges will decrease. When *τ* is large enough, the node addition probability is close to zero. In this case, there is almost no addition of nodes and the number of nodes hardly changes. Meanwhile, the number of the edges gets stable for *P*^(*EA*)^ ≈ *P*^(*A*)^ and *P*^(*ED*)^ ≈ *P*^(*D*)^.

Fig 12(c) and 12(d) shows that the average layer link density increases with parameter *τ*. From Eq (9), we can see that, the node addition probability *P*^(*NA*)^(*t*) is a decreasing function of *τ*. Smaller *τ* corresponds to relatively larger *P*^(*NA*)^(*t*). Thus more new enterprises enter the supply chain, making the linkage of the network sparser. Similarly, Larger *τ* corresponds to relatively smaller *P*^(*NA*)^(*t*). In this case, few new enterprises can enter the supply chain, thus existing enterprises have to develop more partnerships among themselves, making the linkage of the network denser and the average strength stronger.

From the above analysis, we could find that smaller *η* and *μ*, and larger *τ* are suitable for the supply chain with low development rate, otherwise, they are for that with fast rate.

Of course, if the time series data of the supply chain could be obtained, we could get the optimal parameters for the external market demand and internal competition-cooperation in our evolution model, which can describe development process of the supply chain closely. During the optimal parameters searching, we should first combine the information of structure property and industry background of the supply chain, analyze its priori information, and then apply heuristic algorithm or grid search based method to find the optimal parameters.

### Benchmark data validation

*Ref*. [48] introduces 38 datasets of supply chain networks from different fields, including Industrial Organic Chemicals, Semiconductors and Related Devices, Computer Peripheral Equipment, Food Preparations, Cutlery and others. They are collected from actual business partnerships of 29 companies, in which, although some specific data are reprocessed for the protection of company confidentiality, their topologies are preserved. These datasets could be seen in S2 File. The scales of these networks are heterogeneous, with the number of nodes ranging from 8 to 2025 and the number of edges ranging from 10 to 16225. Although these supply chains cover various industries, they all includes some manufactures, whose numbers are from two to 687. Thus, these 38 supply chain data can be regarded as benchmark data for supply chain area [49–51].

**Fig 13:**
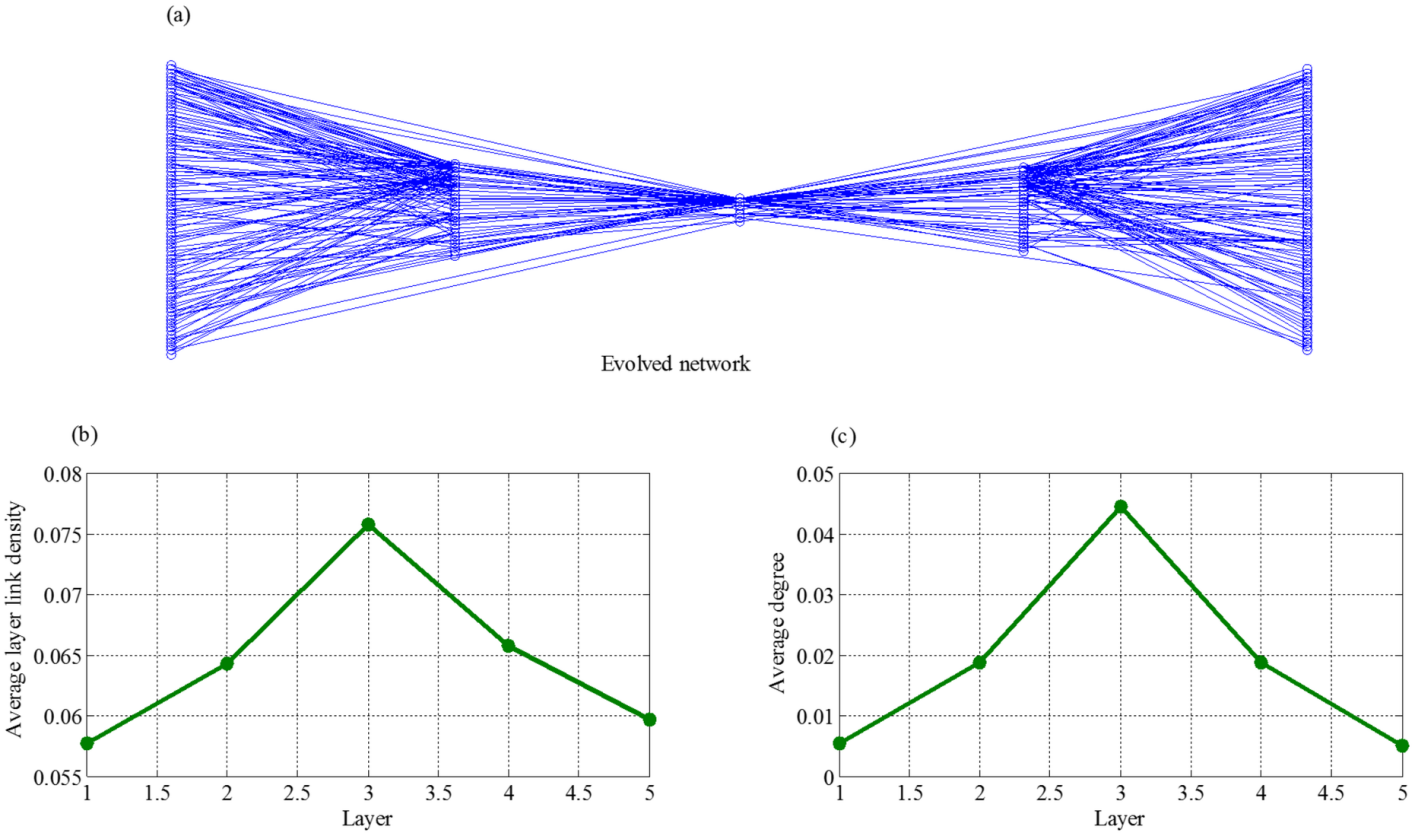
Topology features of networks obtained by our evolution model, where (a) is an example of supply chain networks constructed by our model, (b) is average layer link density of different layers and (c) is average strength of different layers.

Here we use these 38 real supply chain networks to evaluate our evolution model. We construct supply chain networks with the same layers as the real supply chain network by performing our model for 300 steps. Then we compare the topologies of the constructed networks with those of the real networks. The simulation results shows that, no matter how we adjust the parameters of evolution model in reasonable intervals, all the final evolved supply chains with the same layers have the similar topological structures. Fig 13 shows the topological structure of the supply chain with five layers. In Fig 13, we find that the manufacture layer has highest average layer link density and average strengths. In addition, the farther a layer to the manufacture layer, the smaller these two values.

In order to quantitatively analyze pictorial similarities of average layer link densities between real and simulated network, we calculate the correlation coefficient (CC) of their average layer link densities. The same implement to average strengths. Then the average values of the CC of average layer link density and CC are calculated. The detail information of them could be seen in Table 3 and Table A in S1 File.

**Table 3:** The CC between real and simulated network for ten top-ranked data.

No.	Layer number	CC of average layer link density	CC of average strength	Average value	Rank
**6**	**4**	**0.988104**	**0.952433**	**0.970268**	**1**
1	3	0.966395	0.919543	0.942969	2
**23**	**3**	**0.983844**	**0.875278**	**0.929561**	**3**
**11**	**5**	**0.877931**	**0.968609**	**0.92327**	**4**
**7**	**4**	**0.871512**	**0.967737**	**0.919624**	**5**
31	4	0.859082	0.969858	0.91447	6
17	5	0.867504	0.908695	0.888099	7
**27**	**5**	**0.854568**	**0.872764**	**0.863666**	**8**
**8**	**8**	**0.901541**	**0.800091**	**0.850816**	**9**
**35**	**6**	**0.863714**	**0.785048**	**0.824381**	**10**

**Fig 14:**
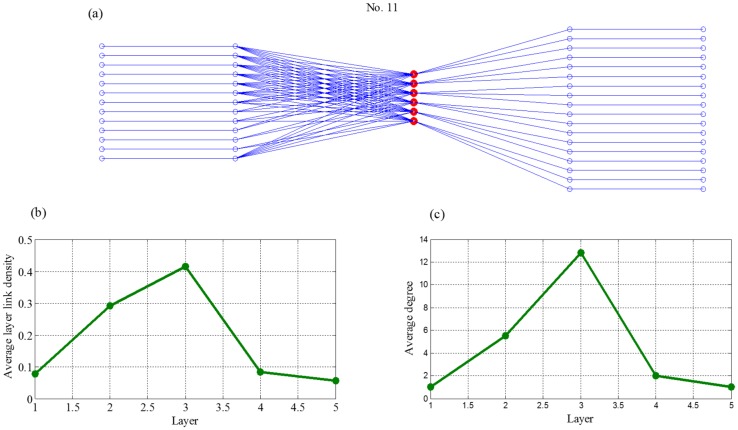
Topology features of network No. 11, where (a) is an example of supply chain network No.11, (b) is average layer link density of different layers and (c) is average strength of different layers.

**Fig 15:**
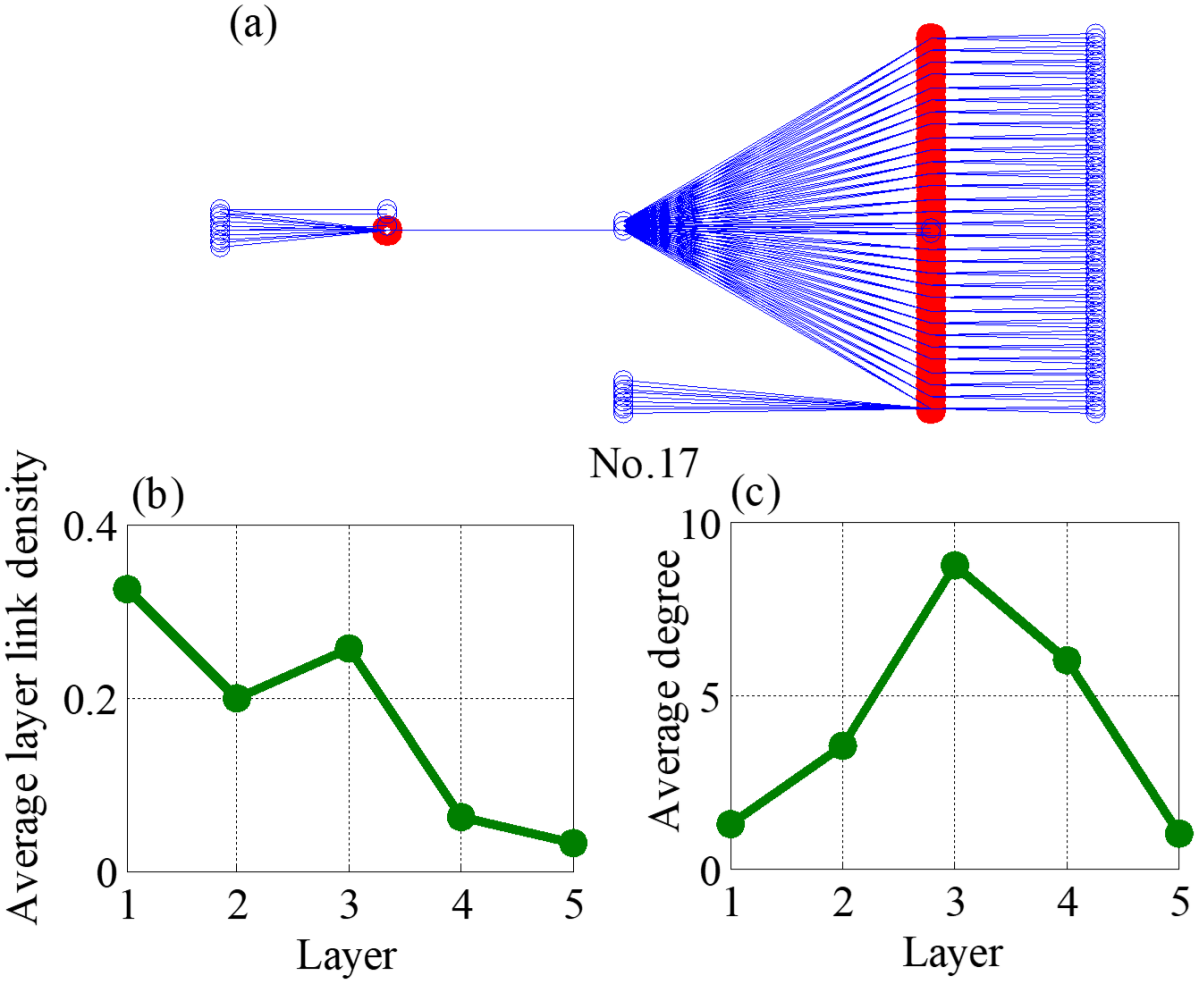
Topology features of network No. 17, where (a) is an example of supply chain network No.17, (b) is average layer link density of different layers and (c) is average strength of different layers.

**Fig 16:**
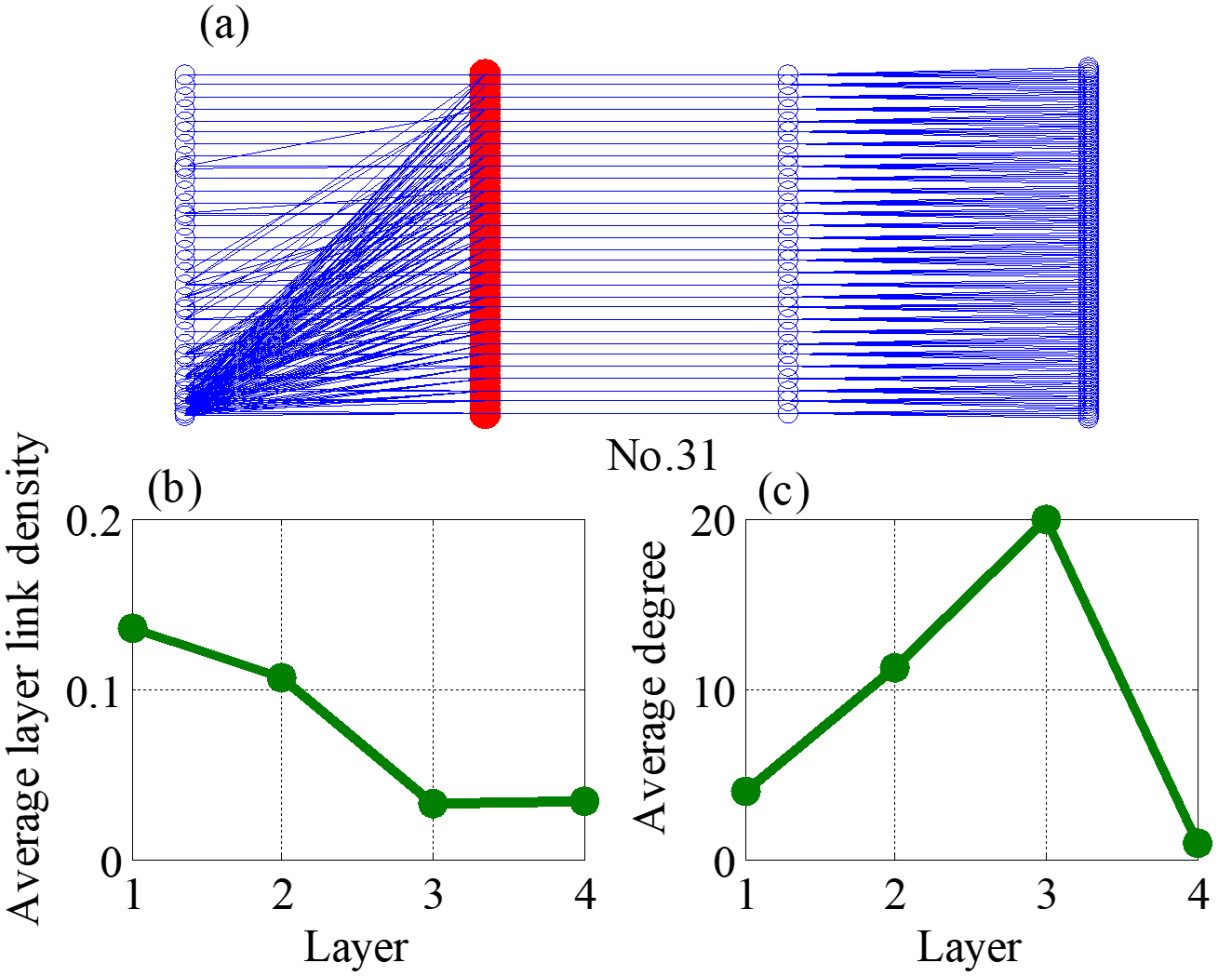
Topology features of network No. 31, where (a) is an example of supply chain network No.31, (b) is average layer link density of different layers and (c) is average strength of different layers.

Theoretically, the closer the value to one, the more similar the pictorial of two supply chains. According to average values of the CC in the Table 3 and the detail information of 38 datasets, eight supply chains are with manufactures as the core from the top ten. They are No.1, No.6, No.7, No.8, No.11, No.23, No.27, and No.35, which are mainly related to construction machinery and equipment, electro-medical and electrotherapeutic apparatus, and power-driven handtool. They are shown in Fig 14 and Figures C-I in S1 File. Although No.17 and No.31 (Figs 15 and 16) are not supply chains with manufactures as the core, they also exist core layers, which are all layer three, but the enterprises in these two layers are not manufactures. It is worth noting that, although manufactures may locates in multiple layers (such as networks No. 6, 8, and 35 in the Appendix), they only have one core layer. For the rest of supply chains, whose topological structures do not match the network evolved by our model, there are two reasons could be explained. First is that the node enterprises in core layer do not belong manufactures, second is that the node number in each layer do not own the dumbbell shaped feature, which is large at both ends but less in the middle.

The simulations suggest that our model can construct networks that have similar topologies as real supply chain networks. Meanwhile, we can see that the distribution pattern of layer link density and average strengths of layers are consistent with that of the networks evolved by our model.

## Conclusions and prospects

External market demand and internal competition-cooperation are the main reasons that promote the development of supply chain networks. Building evolution model is usually an effective method to understand and manage the supply chain system. To overcome the shortages of previous models, which cannot describe the law of supply chain development, we propose a new evolution model under external market demand and internal competition-cooperation. We first give some definitions which are associated with layer-layer association probability. Based on the above definitions, we propose five evolution rules. Then the evolution model is built according these rules. The factors that have not been considered in existing models, including specific topology of supply chain, external market demand, ecological growth and flow conservation, are considered in our model. Finally, the simulation and validation results suggest that the network evolved by our model is similar to real hierarchical supply chain with manufactures as the cores.

In the future, the first worthwhile work is that we will try to collect the time series data of the supply chain, and optimize parameters of the evolution model which is used to describe development process of the supply chain closely. During the optimal parameters searching, we should first combine the information of structure property and industry background of the supply chain, analyze its priori information, and then apply heuristic algorithm or grid search based method to find the optimal parameters. Moreover, we will put the effect of the competition among supply chains to the evolution model, and study its evolution law through the theory of multi-layer hyper network. Additionally, we will refer literature [52–54], then add the study of the invulnerability to our model, and explore its anti-risk ability. Furthermore, we will focus on the research about the impact of external environmental factors on the evolution of supply chain, considering mutation feature of some factors.

## Supporting information

S1 File Supporting information, which includes nine figures and one table. (DOCX)

S2 File The detail information of 38 supply chain datasets, the main contents could be seen in the introduction of the file. (XLS)
