# Correction: Network evolution model for supply chain with manufactures as the core

**DOI:** 10.1371/journal.pone.0196036

**Published:** 2018-04-13

**Authors:** The PLOS ONE staff

The Funding section is incorrect. The correct funding information is as follows: This study was supported by the National Natural Science Foundation of China (CN) grant 61372194 to JZ; the National Natural Science Foundation of China (CN) grant 70871119 to DJ; the National Natural Science Foundation of China (CN) grant 21377166 to TY; and the National Natural Science Foundation of China (CN) grant 61502520. The funders did not have any additional role in the study design, data collection and analysis, decision to publish, or preparation of the manuscript. The publisher apologizes for the error.
