# Supplementary material for: Network evolution model for supply chain with manufactures as the core
Source: PLoS One. 2018 Jan 25;13(1):e0191180. doi: 10.1371/journal.pone.0191180 (PMC5784947; doi:10.1371/journal.pone.0191180)
Supplement: S1 File — (DOCX) [file pone.0191180.s001.docx]

**Supplementary file for “Network Evolution Model for Supply Chain with Manufactures as the Core”**

**Contents**

**Figure A.** **The trends of four topological indices of evolving supply chain network with the change of $\alpha_{c}$.**

**Figure B. The trends of four topological indices of evolving supply chain network with the change of $\alpha_{w}$.**

**Figure C. Topology features of network No. 1.**

**Figure D. Topology features of network No. 6.**

**Figure E. Topology features of network No. 7.**

**Figure F. Topology features of network No. 8.**

**Figure G. Topology features of network No. 23.**

**Figure H. Topology features of network No. 27.**

**Figure I. Topology features of network No. 35.**

**Table A. The CC between real and simulated network.**

### Figure A

Fig. S1 The trends of four topological indices of evolving supply chain network with the change of $\alpha_{c}$. (a) node number, (b) edge number, (c) average layer link density, (d) average strength.

### Figure B

Fig. S2 The trends of four topological indices of evolving supply chain network with the change of $\alpha_{w}$. (a) node number, (b) edge number, (c) average layer link density, (d) average strength.

### Figure C

Topology features of network No.1, where (a) is an example of supply chain network No.1, (b) is average layer link density of different layers and (c) is average strength of different layers.

### Figure D

Topology features of network No. 6, where (a) is an example of supply chain network No.6, (b) is average layer link density of different layers and (c) is average strength of different layers.

### Figure E

Topology features of network No. 7, where (a) is an example of supply chain network No.7, (b) is average layer link density of different layers and (c) is average strength of different layers.

### Figure F

Topology features of network No. 8, where (a) is an example of supply chain network No.8, (b) is average layer link density of different layers and (c) is average strength of different layers.

### Figure G

Topology features of network No. 23, where (a) is an example of supply chain network No.23, (b) is average layer link density of different layers and (c) is average strength of different layers.

### Figure H

Topology features of network No. 27, where (a) is an example of supply chain network No.27, (b) is average layer link density of different layers and (c) is average strength of different layers.

### Figure I

Topology features of network No. 35, where (a) is an example of supply chain network No.35, (b) is average layer link density of different layers and (c) is average strength of different layers.

### Table A

| No | Layer number | CC of average layer link density | CC of average strength | Average value |
| --- | --- | --- | --- | --- |
| 6 | 4 | 0.988104 | 0.952433 | 0.970268 |
| 1 | 3 | 0.966395 | 0.919543 | 0.942969 |
| 23 | 3 | 0.983844 | 0.875278 | 0.929561 |
| 11 | 5 | 0.877931 | 0.968609 | 0.92327 |
| 7 | 4 | 0.871512 | 0.967737 | 0.919624 |
| 31 | 4 | 0.859082 | 0.969858 | 0.91447 |
| 17 | 5 | 0.867504 | 0.908695 | 0.888099 |
| 27 | 5 | 0.854568 | 0.872764 | 0.863666 |
| 8 | 8 | 0.901541 | 0.800091 | 0.850816 |
| 35 | 6 | 0.863714 | 0.785048 | 0.824381 |
| 25 | 3 | 0.896051 | 0.746416 | 0.821234 |
| 16 | 7 | 0.868134 | 0.748417 | 0.808275 |
| 3 | 5 | 0.967173 | 0.640974 | 0.804073 |
| 22 | 9 | 0.660835 | 0.890682 | 0.775758 |
| 24 | 6 | 0.762404 | 0.778633 | 0.770519 |
| 15 | 4 | 0.798385 | 0.711527 | 0.754956 |
| 2 | 4 | 0.832776 | 0.633649 | 0.733213 |
| 37 | 4 | 0.71214 | 0.739866 | 0.726003 |
| 21 | 7 | 0.850112 | 0.526406 | 0.688259 |
| 32 | 5 | 0.889217 | 0.480743 | 0.68498 |
| 36 | 4 | 0.787434 | 0.573701 | 0.680567 |
| 38 | 4 | 0.68865 | 0.671429 | 0.68004 |
| 5 | 5 | 0.883019 | 0.459566 | 0.671293 |
| 4 | 4 | 0.771331 | 0.554128 | 0.662729 |
| 9 | 5 | 0.976919 | 0.341973 | 0.659446 |
| 18 | 8 | 0.580112 | 0.732069 | 0.656091 |
| 12 | 4 | 0.913967 | 0.391717 | 0.652842 |
| 33 | 8 | 0.740292 | 0.551277 | 0.645785 |
| 29 | 5 | 0.870182 | 0.4189 | 0.644541 |
| 14 | 4 | 0.772758 | 0.431289 | 0.602023 |
| 28 | 8 | 0.70869 | 0.466007 | 0.587349 |
| 20 | 10 | 0.741174 | 0.257299 | 0.499236 |
| 34 | 3 | 0.864272 | 0.073906 | 0.469089 |
| 19 | 9 | 0.602611 | 0.303524 | 0.453068 |
| 26 | 6 | 0.500169 | 0.093934 | 0.297052 |
| 30 | 5 | 0.413127 | 0.093925 | 0.253526 |
| 10 | 2 | 0.999652 | 0.884293 | NULL |
| 13 | 2 | 0.999652 | 0.786693 | NULL |
